# ProQ-dependent activation of *Salmonella* virulence genes mediated by post-transcriptional control of PhoP synthesis

**DOI:** 10.1128/msphere.00018-24

**Published:** 2024-02-27

**Authors:** Sofia Bergman, Liis Andresen, Jonas Kjellin, Yolanda Martinez Burgo, Petra Geiser, Sophie Baars, Fredrik Söderbom, Mikael E. Sellin, Erik Holmqvist

**Affiliations:** 1 Department of Cell and Molecular Biology, Biomedical Center, Uppsala University, Uppsala, Sweden; 2 Department of Medical Biochemistry and Microbiology, Science for Life Laboratory, Uppsala University, Uppsala, Sweden; University of Michigan, USA

**Keywords:** ProQ, CLIP-seq, RNA-binding protein, small RNA, *Salmonella*, virulence, PhoP

## Abstract

Gastrointestinal disease caused by *Salmonella enterica* is associated with the pathogen’s ability to replicate within epithelial cells and macrophages. Upon host cell entry, the bacteria express a type-three secretion system encoded within *Salmonella* pathogenicity island 2, through which host-manipulating effector proteins are secreted to establish a stable intracellular niche. Transcription of this intracellular virulence program is activated by the PhoPQ two-component system that senses the low pH and the reduced magnesium concentration of host cell vacuoles. In addition to transcriptional control, *Salmonella* commonly employ RNA-binding proteins (RBPs) and small regulatory RNAs (sRNAs) to regulate gene expression at the post-transcriptional level. ProQ is a globally acting RBP in *Salmonella* that promotes expression of the intracellular virulence program, but its RNA repertoire has previously been characterized only under standard laboratory growth conditions. Here, we provide a high-resolution ProQ interactome during conditions mimicking the environment of the *Salmonella*-containing vacuole (SCV), revealing hundreds of previously unknown ProQ binding sites in sRNAs and mRNA 3′UTRs. ProQ positively affected both the levels and the stability of many sRNA ligands, some of which were previously shown to associate with the well-studied and infection-relevant RBP Hfq. We further show that ProQ activates the expression of PhoP at the post-transcriptional level, which, in turn, leads to upregulation of the intracellular virulence program.

**IMPORTANCE:**

*Salmonella enterica* is a major pathogen responsible for foodborne gastroenteritis, and a leading model organism for genetic and molecular studies of bacterial virulence mechanisms. One key trait of this pathogen is the ability to survive within infected host cells. During infection, the bacteria employ a type three secretion system that deliver effector proteins to target and manipulate host cell processes. The transcriptional regulation of this virulence program is well understood. By contrast, the factors and mechanisms operating at the post-transcriptional level to control virulence gene expression are less clear. In this study, we have charted the global RNA ligand repertoire of the RNA-binding protein ProQ during *in vitro* conditions mimicking the host cell environment. This identified hundreds of binding sites and revealed ProQ-dependent stabilization of intracellular-specific small RNAs. Importantly, we show that ProQ post-transcriptionally activates the expression of PhoP, a master transcriptional activator of intracellular virulence in *Salmonella*.

## INTRODUCTION

During infection of an animal host, bacterial pathogens are challenged by ever-changing environmental conditions, host defense systems, competition with the resident microbiota, and assault by bacteriophages. Consequently, these pathogens are equipped with dedicated systems that promote survival and proliferation during infection. For instance, survival of *Salmonella enterica* serovar Typhimurium (henceforth: *Salmonella*) within host cells is highly dependent on virulence genes encoded in the pathogenicity island 2 (SPI2), and SPI2-associated virulence genes encoded elsewhere on the chromosome (1). The expression of these genes is subject to extensive regulation to ensure appropriate spatial and temporal induction. At the top of this regulatory hierarchy is the two-component system PhoPQ. Once *Salmonella* is located in the *Salmonella*-containing vacuole (SCV), the sensor kinase PhoQ responds to environmental cues such as low Mg^2+^ levels and acidic pH by phosphorylating the response regulator and master transcriptional activator PhoP. PhoP-P, in turn, activates a large number of genes required for intracellular survival and proliferation (2, 3). These include the *ssrAB* operon, a SPI2-encoded two-component system that promotes expression of the SPI2 type-three secretion system (T3SS) (4, 5). In addition, PhoP promotes expression of the SPI2-activating transcription factor (TF) SlyA, which, in turn, stimulates transcription of many virulence genes (6–8). Mechanistically, this often occurs by PhoP- and/or SlyA-dependent displacement of the global DNA-binding protein H-NS (9, 10).

In addition to this well-described transcriptional regulation, an intricate layer of post-transcriptional control seems to govern the intracellular stage of *Salmonella* infection. DualRNA-seq analysis during host cell infection (11), as well as transcriptomic studies in host cell-mimicking growth medium (12, 13), identified many intracellularly expressed small regulatory RNAs (sRNAs). For instance, the sRNA PinT controls the timing of virulence factor expression by targeting mRNAs encoding regulators and effectors important for both the invasion process and intracellular proliferation, respectively (11, 14, 15). Moreover, genetic inactivation of global RNA-binding proteins (RBPs) such as Hfq, CsrA, and ProQ, all of which interact with numerous sRNAs, impairs *Salmonella* virulence gene expression and infectivity (16–20).

The global RBP ProQ belongs to the ProQ/FinO protein family, members of which are found in many proteobacterial species (21, 22). Global profiling in *Salmonella* and *Escherichia coli* (*E. coli*) has attributed hundreds of RNAs as ProQ ligands (23–26), the majority of which are sRNAs and mRNA 3′UTRs. *Salmonella* lacking ProQ shows reduced SPI2 gene expression during cell culture infection and displays an invasion defect (20). However, the global ProQ ligand repertoire under SPI2-inducing conditions, as well as the basis for ProQ-dependent virulence gene expression, has remained unknown.

In this study, we provide a global analysis of ProQ binding sites in *Salmonella* under conditions that mimic the environment of the SCV. Under these conditions, ProQ preferentially binds to sRNAs and 3′ regions of mRNAs. The ProQ RNA ligand repertoire is significantly different between conditions that mimic the extracellular and vacuolar states, respectively, a pattern largely explained by RNA ligand availability. We identify SPI2-induced sRNAs whose expression and/or stability is promoted by ProQ. Some of these are also well-characterized Hfq ligands. Consistent with previous studies, the absence of ProQ entails significantly reduced expression of virulence genes associated with the intracellular stage of infection. As we show here, this is caused by post-transcriptional and ProQ-dependent control of PhoP translation, which, in turn, extensively impacts transcription of downstream virulence genes.

## RESULTS

### Global identification of ProQ binding sites under SPI2-inducing conditions

**Fig 1:**
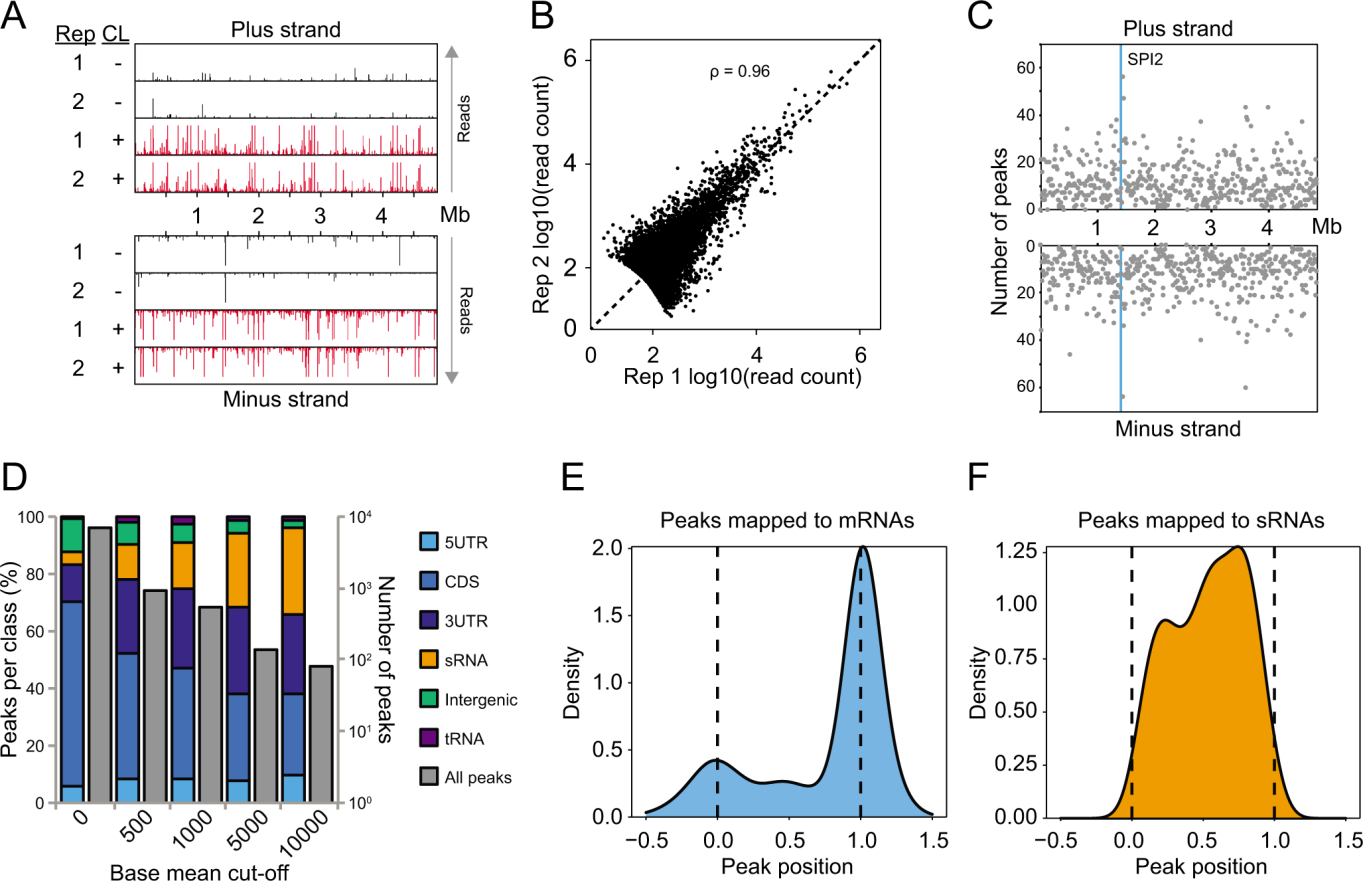
Transcriptome-wide mapping of ProQ binding sites during intracellular-like conditions. (**A**) ProQ CLIP-seq read coverage over the *Salmonella* transcriptome in cross-linked samples and non-crosslinked controls. (**B**) Correlation between two independent ProQ CLIP-seq experiments. Each data point represents normalized read count for a significant (*q* < 0.05) ProQ peak in each of the two experiments. (**C**) Distribution of significant ProQ peaks over the *Salmonella* transcriptome. Each data point represents the number of peaks within a 10,000 window along the genome. (**D**) Number of significant peaks, as well as fraction of peaks per RNA class, as a function of lower normalized read count cut-offs. (**E**) Normalized distribution of ProQ peaks over coding sequences and associated flanking regions. (**F**) Normalized distribution of ProQ peaks in sRNAs. CL, crosslinking; Rep, biological replicate.

Cross-linking and immunoprecipitation followed by sequencing (CLIP-seq) recently identified hundreds of ProQ binding sites in the *Salmonella* transcriptome under standard laboratory conditions (LB medium, early stationary phase) (24). To assess the global ProQ ligand repertoire associated with the intracellular stage of infection, we performed CLIP-seq on *Salmonella* grown in “SPI2 medium”, commonly used to mimic the environment of the SCV (27). Cultures of *Salmonella* carrying a *proQ-3xflag* allele were grown in LB to early exponential phase, shifted to SPI2 medium until reaching an OD_600_ value of 0.3, and thereafter subjected to CLIP-seq using the same protocol as in our previous studies (18, 24). Successful purification of crosslinked and radioactively labeled ProQ-RNA complexes was verified by autoradiographic analysis (Fig. S1). Deep sequencing and mapping of RNA fragments crosslinked to ProQ resulted in strongly enriched read counts in crosslinked over non-crosslinked samples throughout the *Salmonella* transcriptome (Fig. 1A). ProQ binding sites were subsequently identified by peak calling. Based on two separate experiments, which showed strong correlation (Fig. 1B), more than 9,000 significant (*q* ≤ 0.05) peaks were identified (Table S1; Fig. 1C). Increasing the stringency further by adding a cut-off for normalized read count indicated that peaks mapping in sRNAs were more robustly detected than those in, e.g., coding sequences (CDSs) (Fig. 1D). Using a read count cut-off of 1,000, yielding a total peak number comparable to that previously obtained in LB, the majority of peaks mapped to sRNAs, mRNA CDSs, and mRNA 3′UTRs (Fig. 1D). Metagene analysis of peaks mapped to mRNAs revealed a strong peak enrichment in the 3′ region (end of CDS/beginning of 3′UTR) (Fig. 1E), while peaks detected in sRNAs were evenly distributed along the gene body (Fig. 1F).

### RNA expression levels impact the ProQ ligand repertoire

**Fig 2:**
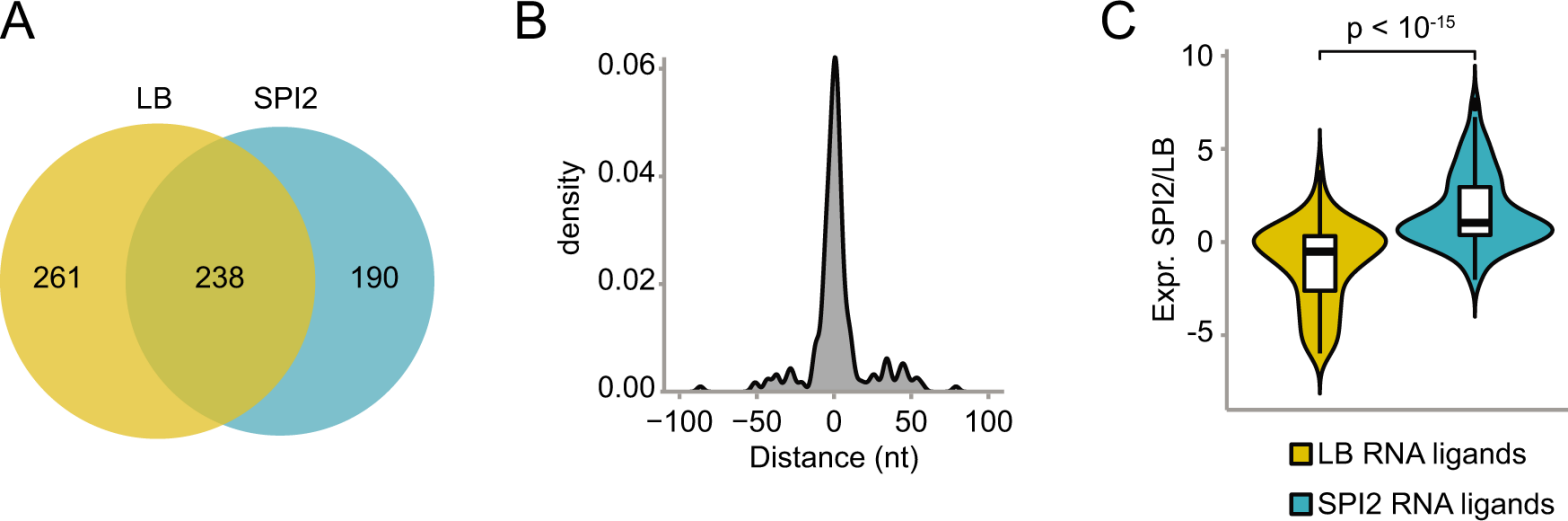
Condition-specific ProQ ligand repertoires. (**A**) Venn diagram showing the overlap between the ProQ interactomes in SPI2-inducing conditions (*q* < 0.05, read count >1,000) and LB medium (24). (**B**) Comparison of ProQ binding site location in ligands commonly found in SPI2-inducing medium and LB. (**C**) Differential expression of RNA ligands uniquely found in SPI2-inducing medium or LB, respectively. Log_2_-transformed expression ratio calculated based on normalized read counts from previously published data (20).

A comparison of the ProQ interactomes obtained in LB (24) and in SPI2 medium (this study) showed that almost half of the LB peaks were also detected in SPI2 medium (Fig. 2A). The peaks detected in both conditions were generally positioned in the same region of the respective RNA ligand; >75% of peaks were positioned less than 20 nucleotides apart (Fig. 2B), indicating that the location of ProQ binding sites in specific RNA ligands is generally consistent between conditions. In addition to the peaks detected in both conditions, a large number of peaks were uniquely detected either in LB or in SPI2 medium. This result may either reflect condition-specific expression of the RNA ligand, or condition-specific binding despite the RNA ligand being present in both growth conditions. To investigate this, the ratio of expression in SPI2 to LB [data from reference (20)] was calculated for each RNA ligand associated with a condition-specific peak. This showed that RNA ligands associated with SPI2-specific peaks are generally expressed at a higher level in SPI2 medium than in LB, and vice versa (Fig. 2C). Hence, ProQ ligand repertoires are strongly correlated with RNA ligand expression levels.

### ProQ impacts the stability of sRNA ligands under SPI2-inducing conditions

**Fig 3:**
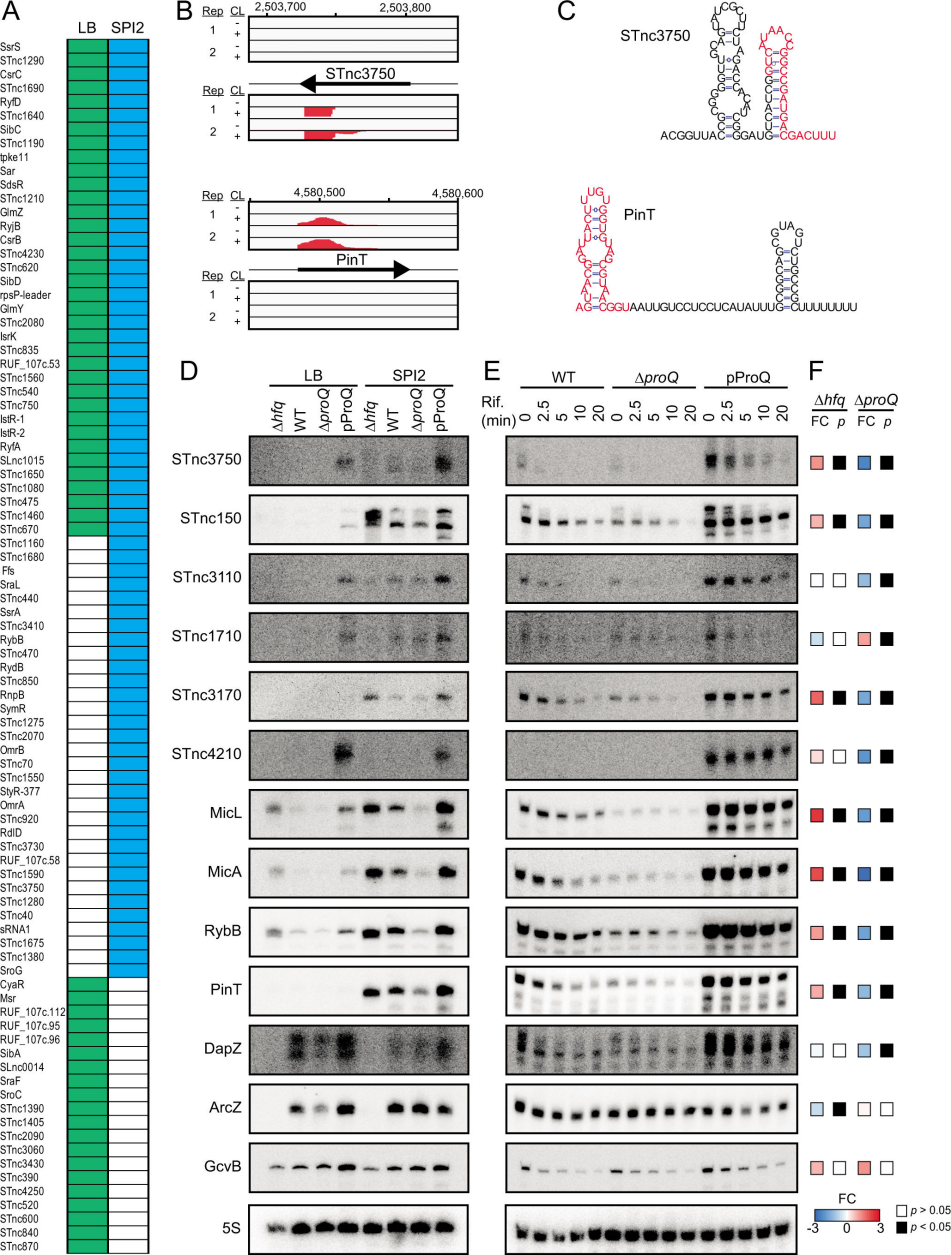
ProQ positively affects the expression and stability of intracellularly induced sRNAs. (**A**) ProQ sRNA ligands detected under SPI2-inducing conditions (*q* < 0.05, read count >1,000) or in LB (24). (**B**) ProQ CLIP-seq read coverage plots for the uncharacterized sRNA STnc3750 and the well-characterized Hfq-dependent sRNA PinT, respectively. Numbers above the plots indicate genome position. (**C**) Secondary structure representations of STnc3750 and PinT based on computational predictions using RNAfold (28). Nucleotides in red denote the ProQ binding site identified by CLIP-seq. (**D**) Steady-state levels of the indicated sRNAs as determined by Northern blot analysis in wild-type, Δ*proQ*, *proQ* complementation (Δ*proQ*, pProQ), and Δ*hfq* strains during growth in LB (early stationary phase) or SPI2-inducing medium (at an OD_600_ of 0.3). (**E**) Northern blot analysis of the indicated sRNAs in wild-type, Δ*proQ*, and *proQ* complementation (Δ*proQ*, pProQ) strains before and after Rifampicin-mediated transcriptional shut-off. (**F**) Differential expression between Δ*proQ* and wild-type, or Δ*hfq* and wild-type strains, respectively, during growth in SPI2-inducing conditions (based on previously published data (20)). Plasmid pProQ expresses ProQ from an IPTG-inducible promoter. The wild-type, Δ*proQ*, and Δ*hfq* strains harbored the empty vector pAR007 (pProQ backbone). FC, log_2_-transformed fold-change. *P,* FDR-adjusted *P*-value. In (**D**) and (**E**), ribosomal RNA 5S served as a loading control.

sRNAs constitute a major class of ProQ ligands in SPI2 medium (Fig. 1), many of which were not identified during growth in LB (24) (Fig. 3A). The SPI2-specific sRNA ligands include both uncharacterized sRNAs such as STnc3750, and well-characterized sRNAs such as PinT (Fig. 3B and C). Using Northern blot analysis, the expression and stability of both uncharacterized sRNAs (STnc150, STnc1710, STnc3110, STnc3170, STnc3750, and STnc4210) and well-characterized sRNAs previously shown to be associated with Hfq (ArcZ, DapZ, GcvB, MicA, MicL, PinT, and RybB) were monitored. The majority of assayed sRNAs were more strongly expressed in SPI2 medium than in LB, showed reduced levels in the absence of ProQ, and could be complemented back to wild-type levels, or even higher, by ProQ complementation from a plasmid (Fig. 3D). In many cases, these effects are consistent with ProQ-dependent changes in sRNA stability (Fig. 3E). Interestingly, during growth in SPI2 medium, levels of the Hfq-associated sRNAs MicA, MicL, PinT, and RybB were strongly reduced in the absence of ProQ, while wild-type levels were observed in a ProQ complementation strain. The absence of Hfq did not decrease but rather increased the abundance of these sRNAs. By contrast, ArcZ, DapZ, and GcvB were strongly reduced in the absence of Hfq but showed less or no ProQ-dependence. These results strongly agree with a reanalysis of previously obtained RNA-seq data (20) (Fig. 3F). Taken together, ProQ promotes the expression levels of many of its sRNA ligands, including both uncharacterized and well-characterized Hfq-associated sRNAs, during growth in SPI2 medium.

### ProQ affects the expression of virulence mRNAs

**Fig 4:**
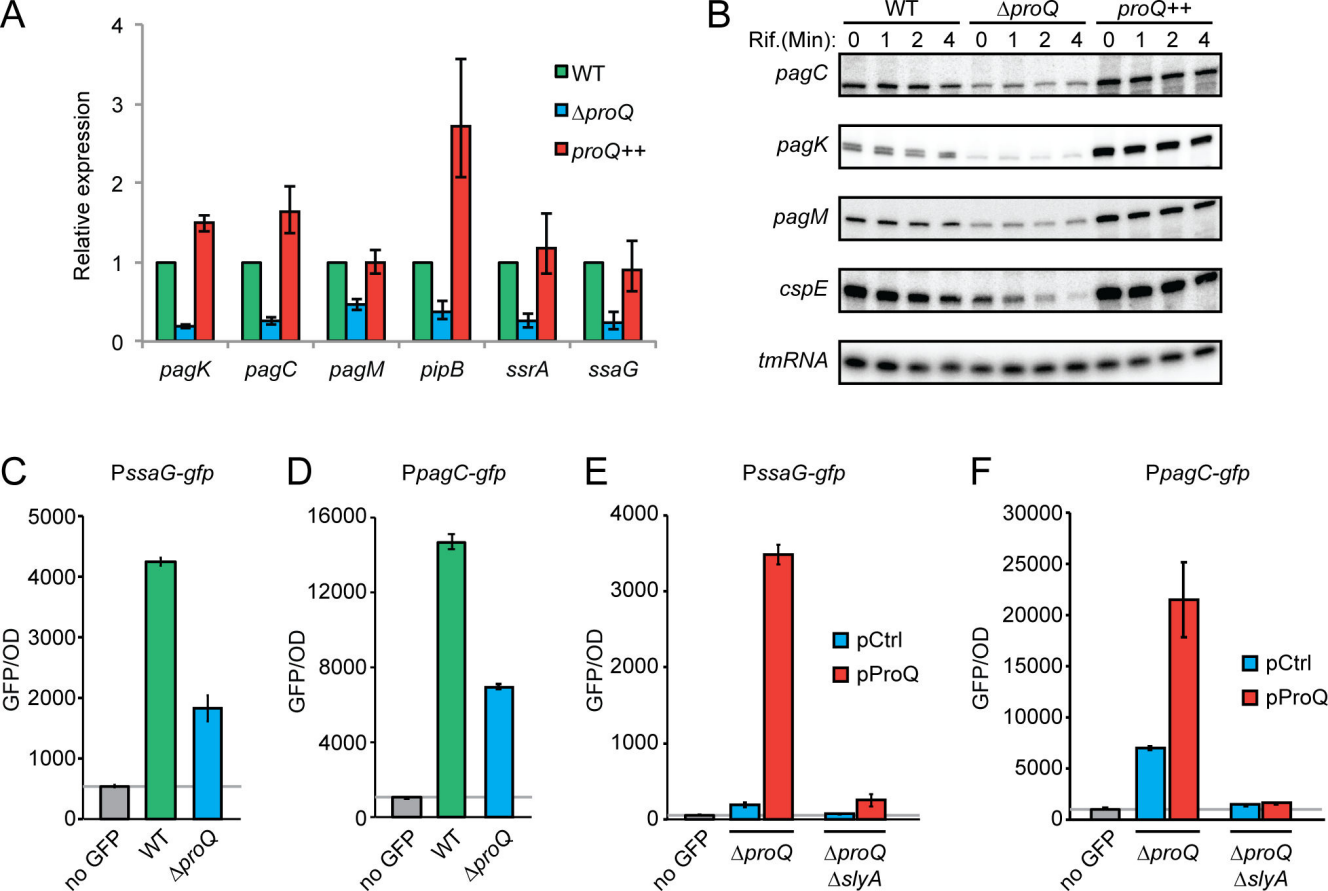
ProQ indirectly promotes transcription of intracellularly induced virulence genes. (**A**) Steady-state levels of the indicated mRNAs in wild-type, Δ*proQ*, and *proQ* complementation strains during growth in SPI2 medium as determined by quantitative real-time PCR (qRT-PCR). (**B**) Northern blot analysis of the indicated mRNAs in wild-type, Δ*proQ*, and *proQ* complementation strains before and after Rifampicin-mediated transcriptional shut-off. The *cspE* mRNA served as a positive control for ProQ-dependent stabilization, while tmRNA served as a loading control. In A and B, *proQ ++* indicates a Δ*proQ* strain expressing *proQ* from its native promoter on a multi-copy plasmid. The wild-type and Δ*proQ* strains harbor the respective empty vector pZE12-luc. (**C–D**) Measurements of GFP expression from transcriptional reporters P*_ssaG_-gfp* (**C**) and P*_pagC_-gfp* (**D**) in wild-type and Δ*proQ* strains during growth in SPI2 medium. (**E–F**) Measurements of GFP expression from transcriptional reporters P*_ssaG_-gfp* (**E**) and P*_pagC_-gfp* (**F**) in Δ*proQ* and Δ*proQ*Δ*slyA* strains with or without ProQ overexpression from an IPTG-inducible promoter on plasmid pProQ during growth in SPI2 medium. In C–F, the wild-type, Δ*proQ*, and Δ*proQ*Δ*slyA* strains harbor the empty vector pAR007 (pProQ backbone).

In addition to sRNAs, the CLIP-seq data indicated possible ProQ-dependent regulation of many virulence-associated mRNAs. These encode structural proteins of the SPI2-encoded T3SS, secreted effectors, virulence-activating TFs, or other virulence-related proteins (Table S1). ProQ’s impact on the expression of SPI2-encoded genes *ssrA* and *ssaG* and other virulence-related genes *pagK*, *pagM*, and *pipB* was monitored by qPCR analysis. All of the tested mRNAs exhibited reduced steady-state levels in a Δ*proQ* strain, and levels similar to a wild-type strain, or even higher, upon complementation by plasmid-expressed ProQ (Fig. 4A). As ProQ promotes the stability of some mRNA ligands (24), mRNA decay rates were monitored by Rifampicin run-off experiments. A positive control, *cspE* mRNA, was clearly destabilized in the absence of ProQ and regained stability upon ProQ complementation (Fig. 4B). By contrast, the decay rate of the tested virulence mRNAs appeared unaffected by ProQ (Fig. 4B), although the steady-state levels followed the pattern observed by qPCR analysis (Fig. 4A). This suggests a ProQ-dependent but indirect effect, presumably at the level of transcription initiation. Indeed, deletion of *proQ* resulted in reduced *ssaG* and *pagC* promoter activity (Fig. 4C and D), while ProQ overexpression strongly increased their expression (Fig. 4E and F). This effect was abolished in strains lacking SlyA (Fig. 4E and F), a transcriptional activator of both *ssaG* and *pagC* (8, 29). We next monitored *ssaG* promoter activity during *Salmonella* infection of human U937 cells by flow cytometry, using the P*_ssaG_-gfp* construct. Deletion of *proQ* reduced the fraction of GFP-expressing cells (Fig. S2A), as well as the mean GFP signal within this fraction (Fig. S2B). Taken together, the absence of ProQ leads to reduced transcription of genes involved in the intracellular stage of infection. Considering that ProQ is known to act at the post-transcriptional level, this effect is likely indirect.

### ProQ affects the expression of *phoP* and *slyA*

**Fig 5:**
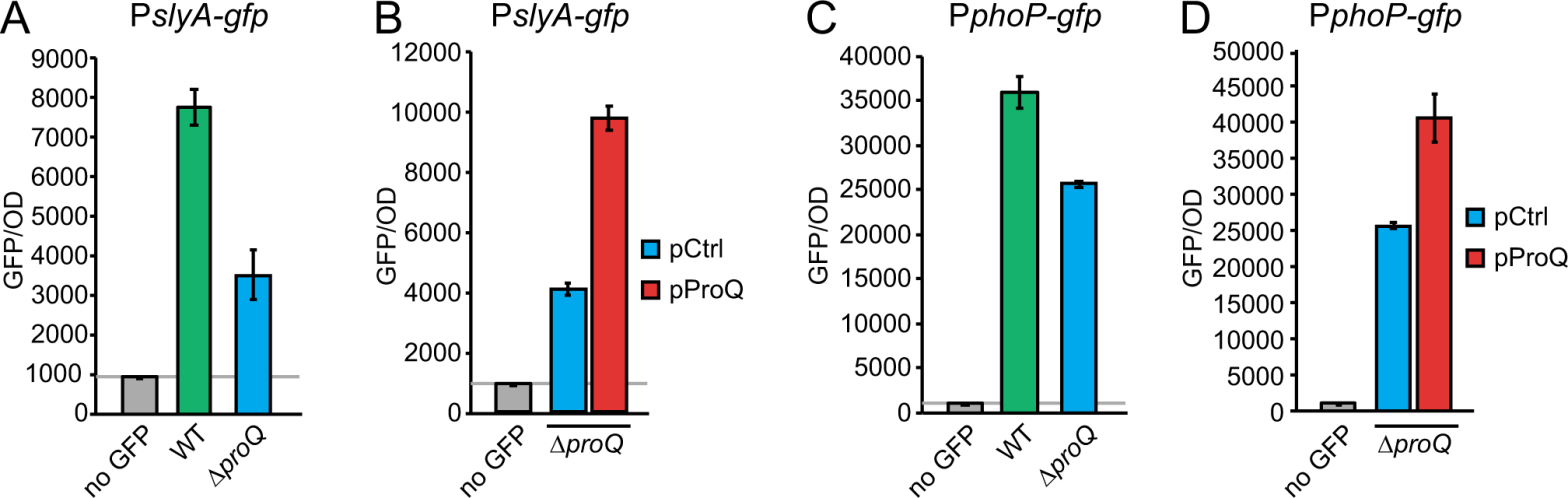
ProQ promotes transcription of *phoP* and *slyA*. Measurements of GFP expression from transcriptional reporters P*_slyA_-gfp* (**A, B**) and P*_phoP_-gfp* (**C, D**) in wild-type and Δ*proQ* strains (**A, C**), or Δ*proQ* with or without ProQ overexpression from an IPTG-inducible promoter on plasmid pProQ (**B, D**), during growth in SPI2 medium. The wild-type and Δ*proQ* strains harbor the empty vector pAR007 (pProQ backbone).

Many intracellularly expressed virulence genes in *Salmonella*, including *ssaG* and *pagC*, are activated by TFs PhoP and SlyA, suggesting that a direct regulatory effect of ProQ, resulting in the observed downstream effects (Fig. 4), may converge at one or both of these regulators. As PhoP and SlyA mutually regulate each other’s expression (7, 30), *slyA* and *phoP* transcription was monitored using promoter-*gfp* fusions. Transcription of both *slyA* and *phoP* decreased in the absence of *proQ* compared to the wild-type strain (Fig. 5A and C), and could be restored by ProQ overexpression (Fig. 5B and D). In accordance, steady-state levels of *slyA* mRNA and SlyA protein were strongly reduced in the absence of ProQ, and restored by ProQ complementation (Fig. S3A and B). However, this is unlikely through effects on mRNA stability, as the *slyA* mRNA decay rate, in contrast to the steady-state level, was unaffected in the *proQ* deletion strain (see Rifampicin experiments; Fig. S3C). Moreover, SlyA protein levels became independent of ProQ when expressed from the heterologous *araBAD* promoter (Fig. S3D). Hence, ProQ appears to indirectly affect expression of *slyA* at the transcriptional level, without effects at the post-transcriptional level.

### ProQ regulates PhoP synthesis at the post-transcriptional level

**Fig 6:**
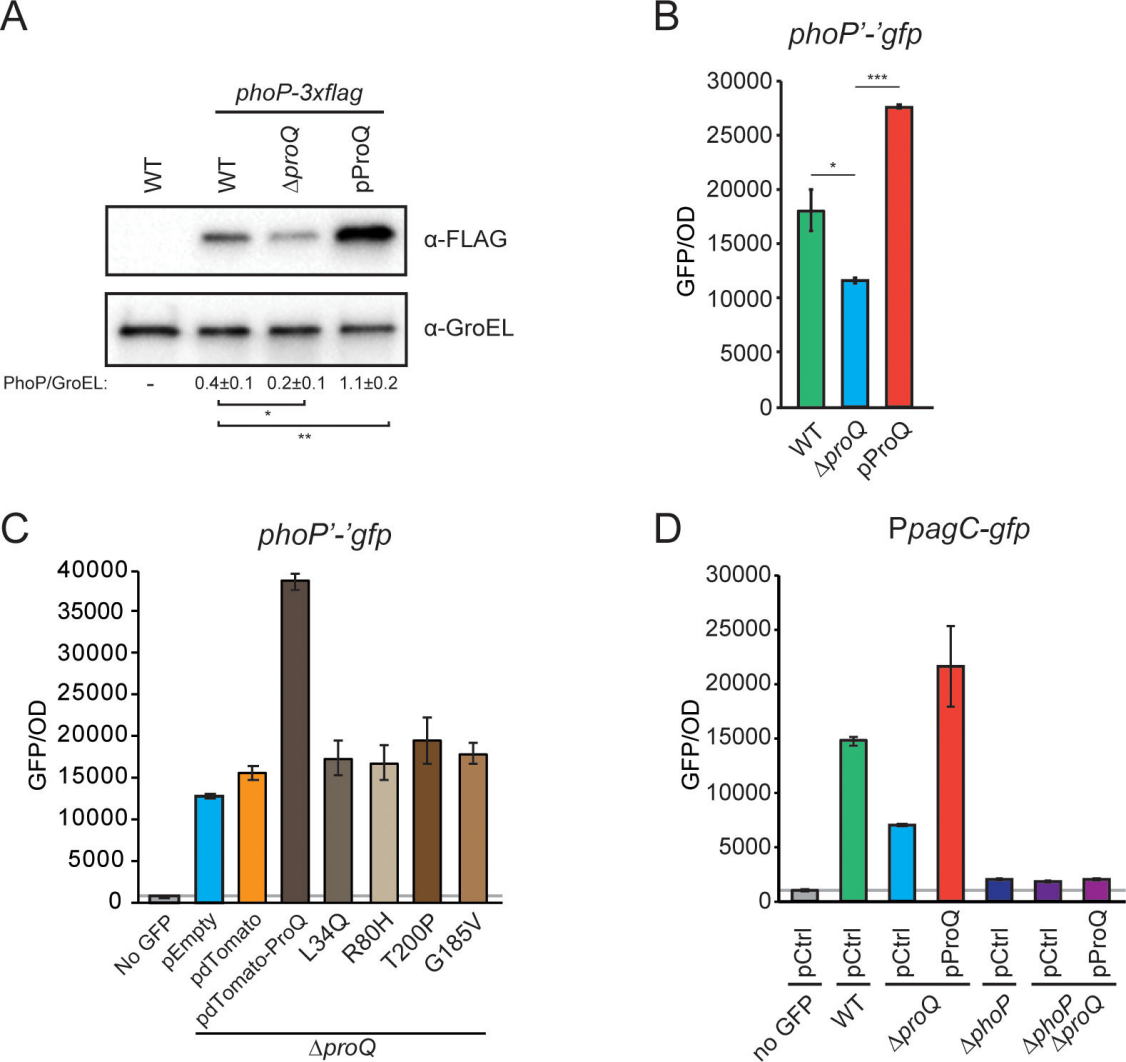
ProQ positively affects translation of PhoP. (**A**) Western blot analysis of chromosomally encoded PhoP-3xFLAG in wild-type, Δ*proQ*, and Δ*proQ* with ProQ overexpression from an IPTG-inducible promoter on a plasmid. GroEL served as a loading control. Numbers below the image represent average values and standard deviation based on three biological replicates. Statistical significance was determined using a two-tailed *t* test (**P < 0.01; *P < 0.05). (**B**) GFP expression from translational *phoP-gfp* fusions in wild-type, Δ*proQ*, and Δ*proQ* with or without ProQ overexpression from an IPTG-inducible promoter on plasmid pProQ during growth in SPI2 medium. Statistical significance was determined using a two-tailed *t* test (***P < 0.001; **P < 0.01; *P < 0.05). (**C**) GFP expression from a translational *phoP-gfp* fusion in a Δ*proQ* strain with ProQ mutants fused to dTomato and expressed from an IPTG-inducible promoter on a plasmid. Control strains include empty vector control (pEmpty), empty vector with dTomato (pdTomato), and wild-type ProQ fused to dTomato (pdTomato-ProQ). Strains were grown in SPI2 medium. (**D**) GFP expression from transcriptional P*_pagC_-gfp* reporter in the indicated strains during growth in SPI2 medium. In all panels, the wild-type, Δ*proQ*, Δ*phoP*, and Δ*phoP*Δ*proQ* strains harbor the empty vector pAR007 (pProQ backbone).

Since ProQ-dependent regulation of SlyA expression appeared to be indirect, we next addressed the possibility of ProQ controlling PhoP expression post-transcriptionally. Western blot analysis showed significantly reduced levels of chromosomally expressed PhoP-3xFLAG upon deletion of *proQ*, and increased levels beyond that of the wild-type strain upon ProQ overexpression (Fig. 6A). Note that attachment of the 3xFLAG tag did not impair PhoP protein activity (Fig. S4). To specifically monitor effects at the post-transcriptional level, a *phoP-gfp* translational fusion was constructed. This encompassed the *phoP* 5′UTR, and the first 10 codons of the *phoP* ORF inserted between a constitutive promoter and *gfp* on a plasmid. Strikingly, deletion of *proQ* resulted in significantly reduced PhoP-GFP translation, while ProQ overexpression increased PhoP-GFP beyond wild-type levels (Fig. 6B). By contrast, a translational *slyA-gfp* fusion was essentially unaffected by altered ProQ levels (Fig. S3E). We next assayed PhoP-GFP translation in strains expressing ProQ mutants identified in recent genetic screens (31–33). Mutation R80H, which completely impairs the RNA-binding activity of the ProQ N-terminal domain (31), abolished ProQ’s ability to activate PhoP translation (Fig. 6C). The same effect was observed with mutation L34Q, which likely impairs the folding of the N-terminal domain (31, 33). Mutations T200P and G185V, both of which impairs the regulatory activity of the ProQ C-terminal domain (32, 33), also completely abolished ProQ-dependent activation of PhoP translation (Fig. 6C). Importantly, the experiments in (Fig. 6C) were carried out with ProQ-dTomato fusion proteins, which do not become unstable upon mutations in the ProQ polypeptide (32, 33). Finally, ProQ-mediated activation of the PhoP-dependent *pagC* promoter was completely abolished in a *phoP* deletion strain (Fig. 6D).

Together these results suggest ProQ to act through a post-transcriptional mechanism to control PhoP translation, which indirectly leads to extensive effects on transcription of PhoP-dependent promoters in the *Salmonella* virulence program.

## DISCUSSION

### A ProQ interactome linked to the intracellular lifestyle of *Salmonella*

In this paper, we have used CLIP-seq to chart the ProQ ligand repertoire in *Salmonella* during conditions that mimic the intracellular environment of eukaryotic host cells. Thousands of ProQ binding sites were detected in different classes of cellular RNAs, primarily in mRNA and sRNA transcripts. Many of these ProQ ligands were not detected in previous studies, emphasizing the importance of conducting global RNA-protein interaction studies across various conditions. By monitoring the steady-state levels and decay rates of sRNA ligands, ProQ binding could be linked to effects on expression for both uncharacterized and previously characterized sRNAs. Among the latter were several well-characterized Hfq-dependent sRNAs. Our analysis of ProQ’s role in the expression of *Salmonella* virulence genes indicates that ProQ positively affects translation of PhoP, which, in turn, leads to enhanced expression of a large number of PhoP-dependent virulence genes.

### ProQ-dependent sRNAs in SPI2-inducing conditions

Performing CLIP-seq under SPI2-inducing conditions identified many sRNAs not previously identified as ProQ ligands, and Northern blot analysis showed that the expression and/or stability of many of these depend on ProQ (Fig. 3). It is important to note that many of these sRNAs are transcriptionally activated by PhoP (13). Thus, ProQ may promote their expression both indirectly through ProQ-dependent activation of PhoP translation and directly by binding and protecting them from degradation.

In line with their strongly elevated expression in SPI2 medium (Fig. 3) (12, 20), these sRNAs are likely to have virulence-related functions. For instance, STnc150 is encoded between the divergently transcribed coding genes *icdA* and *STM1239* and was suggested to promote adhesion and intracellular survival through a so-far unknown mechanism (34). STnc3750 is transcribed from a promoter overlapping the 3′ part of the *pgtE* gene, encoding an outer membrane protease that confers resistance to antimicrobial peptides (35), and is associated with the increased human serum resistance of strain D234580 of *Salmonella* ST313, which causes invasive nontyphoidal Salmonellosis (36). Assigning functions to such uncharacterized sRNAs will be important for fully understanding the post-transcriptional layer of virulence gene regulation in *Salmonella*.

A number of previously verified Hfq-dependent sRNAs crosslinked to ProQ under SPI2-inducing conditions and expression of several of these were dependent on ProQ (Fig. 3). Somewhat unexpected, sRNAs such as MicL, MicA, RybB, and PinT instead showed increased steady-state levels in a strain deleted for *hfq*. However, this result aligns well with a previous RNAseq-based study using the same *Salmonella* strain background and a similar growth medium (20). It thus appears that in some conditions, ProQ becomes the superior RNA chaperone for assuring the expression of a subset of well-characterized Hfq-dependent sRNAs. This highlights the importance of studying RBP and sRNA biology in more physiologically relevant conditions, and suggests that the crosstalk between the ProQ and Hfq interactomes is influenced by the bacterium’s surrounding environment.

### Mechanistic aspects of ProQ-dependent regulation of *phoP* translation

Since the discovery of ProQ as a global RNA-binder in *Salmonella* and *E. coli*, a substantial amount of data regarding ProQ *in vivo* ligands, and ProQ-dependent gene expression, has become available (23–25). A deeper understanding about ProQ’s RNA-binding activity has emerged from biochemical, structural, and mutational studies (31–33, 37–39). By contrast, mechanistic understanding linking ProQ binding events to effects on particular RNA ligands is limited to the study of a few examples. ProQ-dependent mRNA stabilization has been attributed to 3′UTR binding and protection from 3′ to 5′-dependent exonuclease activity (24), while ProQ may protect sRNA-sRNA duplexes from attack by RNase III (25). In line with the ProQ-dependent stabilization of sRNAs observed here (Fig. 3), overexpression of ProQ in *E. coli* resulted in increased RNA stabilization on a global level (25), indicating that protection from ribonuclease attack may be a general function for ProQ. This could be further clarified by systems-level measurements of RNA decay rates with respect to ProQ expression. ProQ also affects sRNA-dependent regulation of mRNA translation: ProQ promotes RaiZ-dependent inhibition of *hupA* mRNA translation (40) and participates in repression of *mgtB* translation by the STnc540 (20). However, the mechanistic details of ProQ’s role in sRNA-dependent regulation are so far not entirely clear.

We show here that ProQ activates expression of PhoP at the post-transcriptional level (Fig. 6). In contrast, ProQ-dependent activation of SlyA, PagC, and SsaG expression appears to be strictly transcriptional (Fig. 4 through 6; Fig. S3) and likely mediated directly or indirectly through PhoP (7, 9, 41). How does ProQ enhance PhoP synthesis mechanistically? The lack of a CLIP-seq peak in the *phoP* 5′UTR (Table S1) argues against a mechanism solely depending on ProQ binding. Tentatively, the *phoP* 5′UTR may be targeted by a yet to be identified sRNA that similar to RaiZ and STnc540 requires ProQ to maintain cellular stability and/or execute target mRNA binding and regulation. In *E. coli*, the two sRNAs MicA and GcvB target the *phoP* mRNA to inhibit translation (42, 43). However, the fact that ProQ enhances the levels of MicA, and has no effect on GcvB levels (Fig. 3D and E), speaks against these sRNAs being responsible for the ProQ-dependent effect on PhoP translation. Global identification of RNA-RNA interactions associated with ProQ using the RIL-seq approach may allow identification of so-far unknown sRNAs targeting the *phoP* mRNA (25).

ProQ’s positive effect on the expression of SPI2 genes and other members of the PhoP regulon fully aligns with a previous study (20). However, in contrast to what we show here, that study did not observe ProQ-dependent effects on PhoP levels in Western blot assays. We do not have a clear explanation for this discrepancy but conclude that our observations of ProQ-dependent effects not only on PhoP protein levels (Western blot analysis, Fig. 6) but also on translation (*phoP-gfp* translational fusion, Fig. 6), and transcription (P*_phoP_-gfp* fusion, Fig. 5) strongly argues for ProQ being a *bona fide* regulator of PhoP expression.

### Outlook

The present study strengthens the notion of ProQ as a major factor in control of the intracellular virulence program of *Salmonella*. In addition, ProQ is known to promote other infection-relevant processes, including flagellar motility and biofilm formation (20, 33, 44). The approach used here lend itself for studying additional ProQ-dependent phenotypes for which the underlying mechanism of regulation is currently unknown.

## MATERIALS AND METHODS

### Bacterial growth conditions

Bacterial cultures were routinely grown aerobically in LB medium at 37°C with shaking at 220 rpm. To mimic the environment of the SCV, bacteria were grown in a minimal SPI2-inducing medium, as previously described (27). The media were supplemented with antibiotics (ampicillin 100 µg/mL, chloramphenicol 30 µg/mL, tetracycline 12.5 µg/mL, and/or kanamycin 50 µg/mL) when appropriate. IPTG (0.5 mM) or arabinose (0.0016%) was added to the media to induce expression of plasmid-borne ProQ or 3xFLAG-SlyA, respectively.

### Strain construction

Bacterial strains and oligonucleotides used in the study are listed in Table S2 and Table S3, respectively. Deletion of *proQ* and *slyA* was achieved by P22 transduction with JVS-11364 (23), and C1892 from the McClelland collection (45), as donor strains, respectively, and wild-type SL1344 (JVS-1574) as the recipient strain. Chromosomal antibiotic cassettes were removed using the pCP20 plasmid (46). Lambda red recombination including a pSim5-tet plasmid (47) was used to construct the FLAG-tagged strains EHS-2294 (*3xflag-slyA*) and EHS-3288 (*phoP-3xflag*). Briefly, the *kan-sacB* cassette was amplified (*slyA*: primers EHO-1363/-1364; *phoP*: primers EHO-1869/-1870), and inserted after the start codon of *slyA* or before the stop codon of *phoP*, respectively. Next, a fragment including the 3xFLAG sequence, either amplified from plasmid pSB002 (EHO-1502/-1503) or generated by Klenow fill-in of annealed partially overlapping primers (EHO-1871/-1872), was used to replace the *kan-sacB* cassette. Replacement of *kan-sacB* cassette was verified by growth on sucrose plates, PCR, and DNA sequencing.

### Plasmid construction

All plasmids used in this study are listed in Table S4. Transcriptional fusions were constructed by inserting PCR-amplified promoter sequences between BamHI and XhoI in plasmid pUA66. The following primers were used to amplify the insert for the respective plasmids, EHO-1314/-1253 (pYMB005), EHO-1315/-1508 (pSB008), EHO-1603/-1604 (pSB011), and EHO-1623/-1624 (pSB012).

To create plasmid pEH809, the MB1 origin of plasmid pBR322 was amplified with primers EHO-1673/-1674, cut with NsiI and XhoI and ligated to a PCR fragment amplified from pBAD33 using primers EHO-1675/-1676. To create plasmid pEH811, a *3xFLAG-slyA* fragment (including 5′ and 3′UTRs) amplified from plasmid pSB001 with primers EHO-1689/-1690 was inserted between the NheI and HindIII sites in pEH809. To create plasmid pEH791, pXG-1 was amplified (EHO-450/-1343) and cut with NsiI and XbaI followed by ligation with a PCR fragment encompassing the *slyA* gene (EHO-1340/-1342). To create pSB001, the pEH791 plasmid was amplified with EHO-1458/-1459 which inserts a 3xFLAG at the N-terminal of the *slyA* gene.

To create translational fusions (pEH839 and pEH843), SL1344 genomic DNA was amplified with EHO-1786/-1787 (*phoP* 5'UTR: from TSS 34 bp upstream start codon to the 10th codon) and EHO-1339/-1790 (*slyA* 5'UTR: from TSS 198 bp upstream start codon to the 10th codon) and cloned in frame with GFP on pXG10-SF using NsiI and NheI.

### CLIP-seq

*Salmonella* SL1344 carrying a chromosomal *proQ-3xflag* allele was grown in 20 mL LB medium to an OD_600_ of 2.0, after which the cells were washed twice with PBS, once with SPI2 medium, and thereafter diluted 1:50 into fresh SPI2 medium. When the culture reached an OD_600_ of 0.3, half of the culture was irradiated with UV light (254 nm, 800 mJ/cm^2^), while the other half was left untreated. Immunoprecipitation, Benzonase treatment, dephosphorylation, radioactive labeling, SDS-PAGE, and RNA purification were carried out as described previously (24). DNA libraries were prepared using the NEBNext Small RNA Library kit (NEB) according to the manufacturer’s instruction and sequenced on an Illumina NextSeq500 instrument at vertis Biotechnologie AG (Freising, Germany).

### CLIP-seq data analysis

Adapter trimming and merging of read pairs was performed with SeqPrep (https://github.com/jstjohn/SeqPrep). Pairs were merged if the resulting read was at least 12 nucleotides long with at least 12 bases overlapping. Read mapping was performed against the *Salmonella* SL1344 chromosome (NC_016810.1) with bowtie 1.2.2 (48) allowing for one mismatch and only reporting the best match for each read. Read coverage was analyzed by converting mapped reads to bigWig format with BEDTools genomecov (49) and bedGraphToBigWig (50). Peak calling was performed with PEAKachu (51). The tool was run in adaptive mode with mad-multiplier (-m) 1.0 and fold change (-f) 1.0 using paired-replicates (-r) of BAM files for the respective pairs of crosslinked and control libraries as input. The maximum fragment size (-M) was set to 25 and annotations in GFF format were used to map overlapping features to called peaks. For normalization, “manual” mode was selected together with size factors calculated as in (24).

### Rifampicin run-off experiments

Bacterial cultures were grown in LB overnight and inoculated 1:100 in fresh LB and grown to an OD_600_ of 2.0. Cultures were then diluted 1:100 in SPI2 medium supplemented with 0.5 mM IPTG and appropriate antibiotics. At an OD_600_ of 0.3–0.4, a sample from the culture (0 min sample) was mixed with 0.2 volumes of stop solution (95% ethanol, 5% phenol) and immediately frozen in liquid nitrogen. Rifampicin was added to the remaining culture at a final concentration of 500 µg/mL, and samples were taken at indicated timepoints, mixed with stop solution, and snap-frozen in liquid nitrogen. Samples were stored at −80°C until RNA extraction.

### RNA extraction

Samples were thawed on ice and centrifuged for 10 min at 13,000 rpm (2 mL tubes) or at 5,000 rpm (15 mL tubes) at 4°C. The supernatant was discarded, and the pellets were resuspended in TE buffer pH 8.0 with 0.5 mg/mL lysozyme. SDS (final concentration 1%) was added, and the tubes were mixed by inversion and incubated for 2 min at 64°C. After the incubation, 0.1 vol of NaOAc 3M (pH 5.2) and 1 vol of acid phenol was added, and the samples were mixed thoroughly. The samples were incubated at 64°C for 6 min and mixed by inversion 6–10 times during the incubation time. The samples were then placed on ice for 5 min and centrifuged for 10 min at 13,000 rpm at room temperature. The aqueous layer was transferred to a Phase-Lock tube, and 1 vol of chloroform was added to each tube. The samples were mixed vigorously by shaking for 30 s and centrifuged 5 min at 13,000 rpm at room temperature. The aqueous layer was transferred to a clean tube and mixed with 3 volumes of ice-cold 30:1 mix (EtOH: 3 M NaOAc pH 5.2) and incubated 12–16 h at −20°C. The samples were then centrifuged for 30 min at 13,000 rpm and 4°C, and the pellets were washed with 80% ice-cold EtOH. After a second centrifugation for 10 min (13,000 rpm, 4°C), the pellets were air dried, dissolved in sterile water, and incubated for 4 min at 65°C with shaking at 900 rpm. RNA quality was assured by agarose gel electrophoresis and SYBR safe (Invitrogen) staining.

### Quantitative real-time PCR

Total RNA samples were DNase-treated with TurboDNase followed by phenol:chlorophorm extraction. DNase-treated total RNA (2 µg) was used for cDNA synthesis using the Maxima H Minus First Strand cDNA Synthesis Kit (#K1652, Thermo Scientific). Synthesized cDNA equivalent to 40 ng total RNA was used in each quantitative real-time PCR (qRT-PCR) with Power SYBR Green PCR Master Mix and analyzed with a Step one Plus real time PCR system (Applied Biosystems). The house-keeping gene *recA* was used for normalization. Primers used for qRT-PCR are listed in Table S2.

### Northern blot

Total RNA samples (5–10 µg) were diluted 1:1 in RNA loading buffer (GLII). The samples were incubated for 3 min at 95°C before loaded on an 6% (vol/vol) polyacrylamide (PAA)/ 8 M urea gel together with a radiolabeled pUC19 MSP1 marker (ThermoFischer). After separation, the RNA was transferred to a nitrocellulose XL membrane (GE Healthcare) or a nitrocellulose Hybond-N+ membrane (Amersham, Cytiva) by wet electroblotting at 4°C overnight. RNA was crosslinked to the membrane by UV light exposure at 1200 mJ. The cross-linked membrane was prehybridized in church buffer (0.5 M sodium phosphate buffer pH 7.2, 1 M EDTA, 7% SDS) for 45 min at 42°C before a 5′-P^32^-labeled DNA oligonucleotide was added to the hybridization buffer and the incubation continued for 12–18 h. The membrane was then washed twice in 2 × SSC/0.1% SDS and one time in 0.5 × SSC/0.1% SDS. The membrane was dried, exposed to a phosphor screen, and scanned on a Typhoon phosphorimager (Cytiva).

### Western blot

Protein samples were harvested from *Salmonella* strains grown in SPI2 media to an OD_600_ of 0.3 or 0.4. The samples were centrifuged for 10 min at 13,000 rpm and 4°C, and the pellets were resuspended in loading buffer (2× Laemmli Sample Buffer, Bio-Rad). Before loading, samples were heated for 4 min at 95°C and cooled on ice. The samples and a size marker (PageRuler Prestained Protein Ladder, Thermo Fisher Scientific) were separated on precast Any kD Mini-PROTEAN TGX Stain-Free Protein Gels (Bio-Rad) and transferred to a PVDF membrane using the Trans-Blot Turbo Mini PVDF Transfer Packs (Bio-Rad) in the Trans-Blot Turbo Transfer Starter System (Bio-Rad) at 2.5 A and 25 V for 3 min. The membrane was blocked with 3% BSA (Sigma) in TBS-T buffer (50 mM Tris, 150 mM NaCl, 0.1% Tween 20, pH 7.4–7.6) overnight at 4°C. The membrane was then rinsed with TBS-T for a few seconds and then incubated with TBS-T for 10 min and probed with an anti-GroEL-Peroxidase Conjugate antibody produced in rabbit 1:50,000 (Sigma), a monoclonal anti-FLAG M2-Peroxidase (HRP) antibody produced in mouse 1:10 000 (Sigma), or an anti-ProQ antibody at a 1:10,000 in TBS-T 3% BSA for 1 h at room temperature. The anti-ProQ antibody was followed by incubation with an HRP-conjugated anti-rabbit antibody (Sigma) at 1:100,000 in TBS-T 3% BSA for 1 h. After incubation with antibodies, the membranes were washed in TBS-T for 10 min three times. The membranes were then developed with ECL detection reagent (GE Healthcare Life Sciences) and scanned in a ChemiDoc MP System (Bio-Rad).

### Growth in 96-well plate reader

Bacterial cultures were grown in LB overnight at 37°C, diluted 1:100 in SPI2 medium, loaded in a 96-well plate, and grown for 16–20 h at 37°C, shaking for 30 s every 10 min in a plate reader (Tecan infinite 200Pro). GFP and OD_600_ were measured every 10 min.

### Monocyte cell line culture

The pro-monocytic cell line U937 (ATCC) was cultured in Roswell Park Memorial Institute (RPMI) 1640 medium (Gibco) supplemented with 10% heat inactivated Fetal Bovine Serum (HI-FBS) (Gibco) and Penicillin-Streptomycin (PenStrep) (Gibco) in a T25 flasks (Sarstedt) at 37°C with 5% CO_2_. Cells were kept at a density of 100,000–1,500,000 cells/mL and passaged every 2–3 days.

### Infection assays

*Salmonella* strains, carrying the P*ssaG*-GFP reporter plasmid as indicated, were grown on LA plates with appropriate antibiotics and subsequently inoculated in four biological replicates in 3 mL LB supplemented with 0.3 M NaCl and kanamycin for 12 h at 37°C on a rotating wheel. At the day of the infection, U937 cells were seeded in a round-bottom 96-well plate with a concentration of 100,000 cells/well. The overnight *Salmonella* cultures were sub-cultured in 3 mL LB with 0.3 M NaCl and incubated at 37°C for 4 h. After sub-culturing, the bacteria were spun down at 12,700 rpm and re-suspended in RPMI medium. The *Salmonella* strains were added to the U937 cells at a multiplicity of infection (MOI) of 16 and incubated at 37°C, 5% CO_2_ for 30 min to allow for invasion. After incubation, the plate was centrifuged at 100 *g* for 1 min, and the cells were resuspended in RPMI supplemented with 100 µg/mL Gentamicin. The cells were incubated at 37°C, 5% CO_2_ for 3.5 h to allow *Salmonella* to undergo intracellular maturation and *ssaG*-driven GFP expression. After the incubation, the samples were prepared for flow cytometry. The cells were centrifuged at 300 *g* for 1 min and washed two times with washing solution (PBS with 1% BSA). The samples were then resuspended in 2% PFA and fixed for 20 min in darkness. After fixation, the samples were washed one more time as described before and dissolved in washing solution. The samples were analyzed with flow cytometry using a MACSQuant VYB (Miltenyi Biotec), measuring 30,000 events, and the data were acquired by the MACSQuantify Software (Miltenyi Biotec). The software tool FlowJo (FlowJo, LLC 2006–2021) was used to analyze the data.

## Data Availability

The ProQ CLIP-seq data is available at the NCBI Gene Expression Omnibus with the accession number GSE250014.

## SUPPLEMENTAL MATERIAL

Supplemental Material msphere.00018-24-s0001.pdf Figure S1-S4; Tables S2-S4.

Table S1 msphere.00018-24-s0002.xlsx CLIP-seq data.

ASM does not own the copyrights to Supplemental Material that may be linked to, or accessed through, an article. The authors have granted ASM a non-exclusive, world-wide license to publish the Supplemental Material files. Please contact the corresponding author directly for reuse.

## References

[1] Jennings E, Thurston TLM, Holden DW. 2017. Salmonella SPI-2 type III secretion system effectors: molecular mechanisms and physiological consequences. Cell Host Microbe 22:217–231. doi:10.1016/j.chom.2017.07.009 28799907

[2] Fàbrega A, Vila J. 2013. Salmonella enterica serovar Typhimurium skills to succeed in the host: virulence and regulation. Clin Microbiol Rev 26:308–341. doi:10.1128/CMR.00066-12 23554419 PMC3623383

[3] Groisman EA, Duprey A, Choi J. 2021. How the PhoP/PhoQ system controls virulence and Mg2+ homeostasis: lessons in signal transduction, pathogenesis, physiology, and evolution. Microbiol Mol Biol Rev 85:e0017620. doi:10.1128/MMBR.00176-20 34191587 PMC8483708

[4] Deiwick J, Nikolaus T, Erdogan S, Hensel M. 1999. Environmental regulation of Salmonella pathogenicity island 2 gene expression. Mol Microbiol 31:1759–1773. doi:10.1046/j.1365-2958.1999.01312.x 10209748

[5] Fass E, Groisman EA. 2009. Control of Salmonella pathogenicity Island-2 gene expression. Curr Opin Microbiol 12:199–204. doi:10.1016/j.mib.2009.01.004 19264535 PMC2805070

[6] Norte VA, Stapleton MR, Green J. 2003. PhoP-responsive expression of the Salmonella enterica serovar typhimurium slyA gene. J Bacteriol 185:3508–3514. doi:10.1128/JB.185.12.3508-3514.2003 12775687 PMC156224

[7] Shi Y, Latifi T, Cromie MJ, Groisman EA. 2004. Transcriptional control of the antimicrobial peptide resistance ugtL gene by the Salmonella PhoP and SlyA regulatory proteins. J Biol Chem 279:38618–38625. doi:10.1074/jbc.M406149200 15208313

[8] Navarre WW, Halsey TA, Walthers D, Frye J, McClelland M, Potter JL, Kenney LJ, Gunn JS, Fang FC, Libby SJ. 2005. Co-regulation of Salmonella enterica genes required for virulence and resistance to antimicrobial peptides by SlyA and PhoP/PhoQ. Mol Microbiol 56:492–508. doi:10.1111/j.1365-2958.2005.04553.x 15813739

[9] Christian Perez J, Latifi T, Groisman EA. 2008. Overcoming H-NS-mediated transcriptional silencing of horizontally acquired genes by the PhoP and SlyA proteins in Salmonella enterica. J Biol Chem 283:10773–10783. doi:10.1074/jbc.M709843200 18270203 PMC2447644

[10] Will WR, Bale DH, Reid PJ, Libby SJ, Fang FC. 2014. Evolutionary expansion of a regulatory network by counter-silencing. Nat Commun 5:1–12. doi:10.1038/ncomms6270 PMC4215172 25348042

[11] Westermann AJ, Förstner KU, Amman F, Barquist L, Chao Y, Schulte LN, Müller L, Reinhardt R, Stadler PF, Vogel J. 2016. Dual RNA-seq unveils noncoding RNA functions in host-pathogen interactions. Nature 529:496–501. doi:10.1038/nature16547 26789254

[12] Kröger C, Colgan A, Srikumar S, Händler K, Sivasankaran SK, Hammarlöf DL, Canals R, Grissom JE, Conway T, Hokamp K, Hinton JCD. 2013. An infection-relevant transcriptomic compendium for Salmonella enterica serovar typhimurium. Cell Host Microbe 14:683–695. doi:10.1016/j.chom.2013.11.010 24331466

[13] Colgan AM, Kröger C, Diard M, Hardt W-D, Puente JL, Sivasankaran SK, Hokamp K, Hinton JCD. 2016. The impact of 18 ancestral and horizontally-acquired regulatory proteins upon the transcriptome and sRNA landscape of Salmonella enterica serovar typhimurium. PLoS Genet 12:e1006258. doi:10.1371/journal.pgen.1006258 27564394 PMC5001712

[14] Kim K, Palmer AD, Vanderpool CK, Slauch JM. 2019. The small RNA pinT contributes to PhoP-mediated regulation of the Salmonella pathogenicity island 1 type III secretion system in Salmonella enterica serovar typhimurium. J Bacteriol 201:e00312-19. doi:10.1128/JB.00312-19 31262841 PMC6755756

[15] Correia Santos S, Bischler T, Westermann AJ, Vogel J. 2021. MAPS integrates regulation of actin-targeting effector SteC into the virulence control network of Salmonella small RNA pinT. Cell Rep 34:108722. doi:10.1016/j.celrep.2021.108722 33535041

[16] Altier C, Suyemoto M, Lawhon SD. 2000. Regulation of Salmonella enterica serovar typhimurium invasion genes by csrA. Infect Immun 68:6790–6797. doi:10.1128/IAI.68.12.6790-6797.2000 11083797 PMC97782

[17] Sittka A, Pfeiffer V, Tedin K, Vogel J. 2007. The RNA chaperone Hfq is essential for the virulence of Salmonella typhimurium. Mol Microbiol 63:193–217. doi:10.1111/j.1365-2958.2006.05489.x 17163975 PMC1810395

[18] Holmqvist E, Wright PR, Li L, Bischler T, Barquist L, Reinhardt R, Backofen R, Vogel J. 2016. Global RNA recognition patterns of post-transcriptional regulators Hfq and CsrA revealed by UV Crosslinking in vivo. EMBO J 35:991–1011. doi:10.15252/embj.201593360 27044921 PMC5207318

[19] Potts AH, Guo Y, Ahmer BMM, Romeo T. 2019. Role of CsrA in stress responses and metabolism important for Salmonella virulence revealed by integrated transcriptomics. PLoS One 14:e0211430. doi:10.1371/journal.pone.0211430 30682134 PMC6347204

[20] Westermann AJ, Venturini E, Sellin ME, Förstner KU, Hardt WD, Vogel J. 2019. The major RNA-binding protein ProQ impacts virulence gene expression in Salmonella enterica serovar typhimurium. mBio 10:e02504-18. doi:10.1128/mBio.02504-18 30602583 PMC6315103

[21] Holmqvist E, Berggren S, Rizvanovic A. 2020. RNA-binding activity and regulatory functions of the emerging sRNA-binding protein ProQ. Biochim Biophys Acta Gene Regul Mech 1863:194596. doi:10.1016/j.bbagrm.2020.194596 32565402

[22] Liao Z, Smirnov A. 2023. FinO/ProQ-family proteins: an evolutionary perspective. Biosci Rep 43:BSR20220313. doi:10.1042/BSR20220313 36787218 PMC9977716

[23] Smirnov A, Förstner KU, Holmqvist E, Otto A, Günster R, Becher D, Reinhardt R, Vogel J. 2016. Grad-Seq guides the discovery of ProQ as a major small RNA-binding protein. Proc Natl Acad Sci USA 113:11591–11596. doi:10.1073/pnas.1609981113 27671629 PMC5068311

[24] Holmqvist E, Li L, Bischler T, Barquist L, Vogel J. 2018. Global maps of ProQ binding in vivo reveal target recognition via RNA structure and stability control at mRNA 3′ ends. Mol Cell 70:971–982. doi:10.1016/j.molcel.2018.04.017 29804828

[25] Melamed S, Adams PP, Zhang A, Zhang H, Storz G. 2020. RNA-RNA Interactomes of ProQ and Hfq reveal overlapping and competing roles. Mol Cell 77:411–425. doi:10.1016/j.molcel.2019.10.022 31761494 PMC6980735

[26] El Mouali Y, Gerovac M, Mineikaitė R, Vogel J. 2021. In vivo targets of Salmonella FinO include a FinP-like small RNA controlling copy number of a cohabitating plasmid. Nucleic Acids Res 49:5319–5335. doi:10.1093/nar/gkab281 33939833 PMC8136791

[27] Coombes BK, Brown NF, Valdez Y, Brumell JH, Finlay BB. 2004. Expression and secretion of Salmonella pathogenicity island-2 virulence genes in response to acidification exhibit differential requirements of a functional type III secretion apparatus and SsaL. J Biol Chem 279:49804–49815. doi:10.1074/jbc.M404299200 15383528

[28] Lorenz R, Bernhart SH, Höner Zu Siederdissen C, Tafer H, Flamm C, Stadler PF, Hofacker IL. 2011. ViennaRNA package 2.0. Algorithms Mol Biol 6:26. doi:10.1186/1748-7188-6-26 22115189 PMC3319429

[29] Okada N, Oi Y, Takeda-Shitaka M, Kanou K, Umeyama H, Haneda T, Miki T, Hosoya S, Danbara H. 2007. Identification of amino acid residues of Salmonella SlyA that are critical for transcriptional regulation. Microbiology (Reading) 153:548–560. doi:10.1099/mic.0.29259-0 17259627

[30] Song H, Kong W, Weatherspoon N, Qin G, Tyler W, Turk J, Curtiss R, Shi Y. 2008. Modulation of the regulatory activity of bacterial two-component systems by SlyA. J Biol Chem 283:28158–28168. doi:10.1074/jbc.M801058200 18678876 PMC2661387

[31] Pandey S, Gravel CM, Stockert OM, Wang CD, Hegner CL, LeBlanc H, Berry KE. 2020. Genetic identification of the functional surface for RNA binding by Escherichia coli ProQ. Nucleic Acids Res 48:4507–4520. doi:10.1093/nar/gkaa144 32170306 PMC7192607

[32] El Mouali Y, Ponath F, Scharrer V, Wenner N, Hinton JCD, Vogel J. 2021. Scanning mutagenesis of RNA-binding protein ProQ reveals a quality control role for the Lon protease. RNA 27:1512–1527. doi:10.1261/rna.078954.121 34497069 PMC8594473

[33] Rizvanovic A, Kjellin J, Söderbom F, Holmqvist E. 2021. Saturation mutagenesis charts the functional landscape of Salmonella ProQ and reveals a gene regulatory function of its C-terminal domain. Nucleic Acids Res 49:9992–10006. doi:10.1093/nar/gkab721 34450657 PMC8464044

[34] Li J, Li N, Ning C, Guo Y, Ji C, Zhu X, Zhang X, Meng Q, Shang Y, Xiao C, Xia X, Cai X, Qiao J. 2021. sRNA Stnc150 is involved in virulence regulation of Salmonella typhimurium by targeting fimA mRNA. FEMS Microbiol Lett 368:fnab124. doi:10.1093/femsle/fnab124 34543394

[35] Guina T, Yi EC, Wang H, Hackett M, Miller SI. 2000. A PhoP-regulated outer membrane protease of Salmonella enterica serovar typhimurium promotes resistance to alpha-helical antimicrobial peptides. J Bacteriol 182:4077–4086. doi:10.1128/JB.182.14.4077-4086.2000 10869088 PMC94595

[36] Hammarlöf DL, Kröger C, Owen SV, Canals R, Lacharme-Lora L, Wenner N, Schager AE, Wells TJ, Henderson IR, Wigley P, Hokamp K, Feasey NA, Gordon MA, Hinton JCD. 2018. Role of a single noncoding nucleotide in the evolution of an epidemic African clade of Salmonella. Proc Natl Acad Sci USA 115:E2614–E2623. doi:10.1073/pnas.1714718115 29487214 PMC5856525

[37] Stein EM, Kwiatkowska J, Basczok MM, Gravel CM, Berry KE, Olejniczak M. 2020. Determinants of RNA recognition by the FinO domain of the Escherichia coli ProQ protein. Nucleic Acids Res 48:7502–7519. doi:10.1093/nar/gkaa497 32542384 PMC7367173

[38] Stein EM, Wang S, Dailey KG, Gravel CM, Wang S, Olejniczak M, Berry KE. 2023. Biochemical and genetic dissection of the RNA-binding surface of the FinO domain of Escherichia coli ProQ. RNA 29:1772–1791. doi:10.1261/rna.079697.123 37607742 PMC10578477

[39] Gonzalez GM, Hardwick SW, Maslen SL, Skehel JM, Holmqvist E, Vogel J, Bateman A, Luisi BF, Broadhurst RW. 2017. Structure of the Escherichia coli ProQ RNA-binding protein. RNA 23:696–711. doi:10.1261/rna.060343.116 28193673 PMC5393179

[40] Smirnov A, Wang C, Drewry LL, Vogel J. 2017. Molecular mechanism of mRNA repression in trans by a ProQ-dependent small RNA. EMBO J 36:1029–1045. doi:10.15252/embj.201696127 28336682 PMC5391140

[41] Bijlsma JJE, Groisman EA. 2005. The PhoP/PhoQ system controls the intramacrophage type three secretion system of Salmonella enterica. Mol Microbiol 57:85–96. doi:10.1111/j.1365-2958.2005.04668.x 15948951

[42] Coornaert A, Chiaruttini C, Springer M, Guillier M. 2013. Post-transcriptional control of the Escherichia coli PhoQ-PhoP two-component system by multiple sRNAs involves a novel pairing region of GcvB. PLOS Genet 9:e1003156. doi:10.1371/journal.pgen.1003156 23300478 PMC3536696

[43] Coornaert A, Lu A, Mandin P, Springer M, Gottesman S, Guillier M. 2010. MicA sRNA links the PhoP regulon to cell envelope stress. Mol Microbiol 76:467–479. doi:10.1111/j.1365-2958.2010.07115.x 20345657 PMC2925231

[44] Sheidy DT, Zielke RA. 2013. Analysis and expansion of the role of the Escherichia coli protein ProQ. PLoS One 8:e79656. doi:10.1371/journal.pone.0079656 24205389 PMC3808355

[45] Porwollik S, Santiviago CA, Cheng P, Long F, Desai P, Fredlund J, Srikumar S, Silva CA, Chu W, Chen X, Canals R, Reynolds MM, Bogomolnaya L, Shields C, Cui P, Guo J, Zheng Y, Endicott-Yazdani T, Yang H-J, Maple A, Ragoza Y, Blondel CJ, Valenzuela C, Andrews-Polymenis H, McClelland M. 2014. Defined single-gene and multi-gene deletion mutant collections in Salmonella enterica sv typhimurium. PLoS One 9:e99820. doi:10.1371/journal.pone.0099820 25007190 PMC4089911

[46] Cherepanov PP, Wackernagel W. 1995. Gene disruption in Escherichia coli: TcR and KmR cassettes with the option of Flp-catalyzed excision of the antibiotic-resistance determinant. Gene 158:9–14. doi:10.1016/0378-1119(95)00193-a 7789817

[47] Koskiniemi S, Pränting M, Gullberg E, Näsvall J, Andersson DI. 2011. Activation of cryptic aminoglycoside resistance in Salmonella enterica. Mol Microbiol 80:1464–1478. doi:10.1111/j.1365-2958.2011.07657.x 21507083

[48] Langmead B, Trapnell C, Pop M, Salzberg SL. 2009. Ultrafast and memory-efficient alignment of short DNA sequences to the human genome. Genome Biol 10:R25. doi:10.1186/gb-2009-10-3-r25 19261174 PMC2690996

[49] Quinlan AR, Hall IM. 2010. Bedtools: a flexible suite of utilities for comparing genomic features. Bioinformatics 26:841–842. doi:10.1093/bioinformatics/btq033 20110278 PMC2832824

[50] Kent WJ, Zweig AS, Barber G, Hinrichs AS, Karolchik D. 2010. BigWig and BigBed: enabling browsing of large distributed datasets. Bioinformatics 26:2204–2207. doi:10.1093/bioinformatics/btq351 20639541 PMC2922891

[51] Bischler T, Förstner KU, Maticzka D, Wright PR. 2021. PEAKachu: a peak calling tool for CLIP/RIP-Seqdata (V0.2.0). Zenodo. Available from: 10.5281/zenodo.4669966

