# Supplementary material for: ProQ-dependent activation of Salmonella virulence genes mediated by post-transcriptional control of PhoP synthesis
Source: mSphere. 2024 Feb 27;9(3):e00018-24. doi: 10.1128/msphere.00018-24 (PMC10964419; doi:10.1128/msphere.00018-24)
Supplement: Supplemental Material — Figure S1-S4; Tables S2-S4. [file msphere.00018-24-s0001.pdf]

Supplementary material for:

ProQ-dependent activation of *Salmonella* virulence genes mediated by post-transcriptional control of PhoP synthesis

Sofia Bergman[1], Liis Andresen[1], Jonas Kjellin[1], Yolanda Martinez Burgo[1], Petra Geiser[2], Sophie Baars[1], Fredrik Söderbom[1], Mikael E. Sellin[2], and Erik Holmqvist[1*]

[1] Department of Cell and Molecular Biology, Biomedical Center, Uppsala University, Uppsala, Sweden.

[2] Science for Life Laboratory, Department of Medical Biochemistry and Microbiology, Uppsala University, Uppsala, Sweden.

[*]Correspondence: erik.holmqvist@icm.uu.se

Figure S1

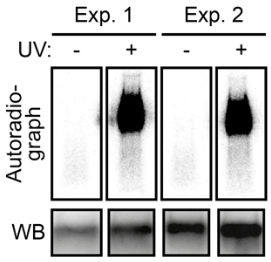

FIG S1. Purification and verification of ProQ-RNA complexes. Autoradiographs of radioactively labeled RNA fragments crosslinked to ProQ (top) and Western blot verification (bottom) after UV crosslinking, immunoprecipitation, gel electrophoresis, and membrane transfer. Exp.1 and Exp. 2 represent two independent experiments. UV: ultraviolet light.

Figure S2

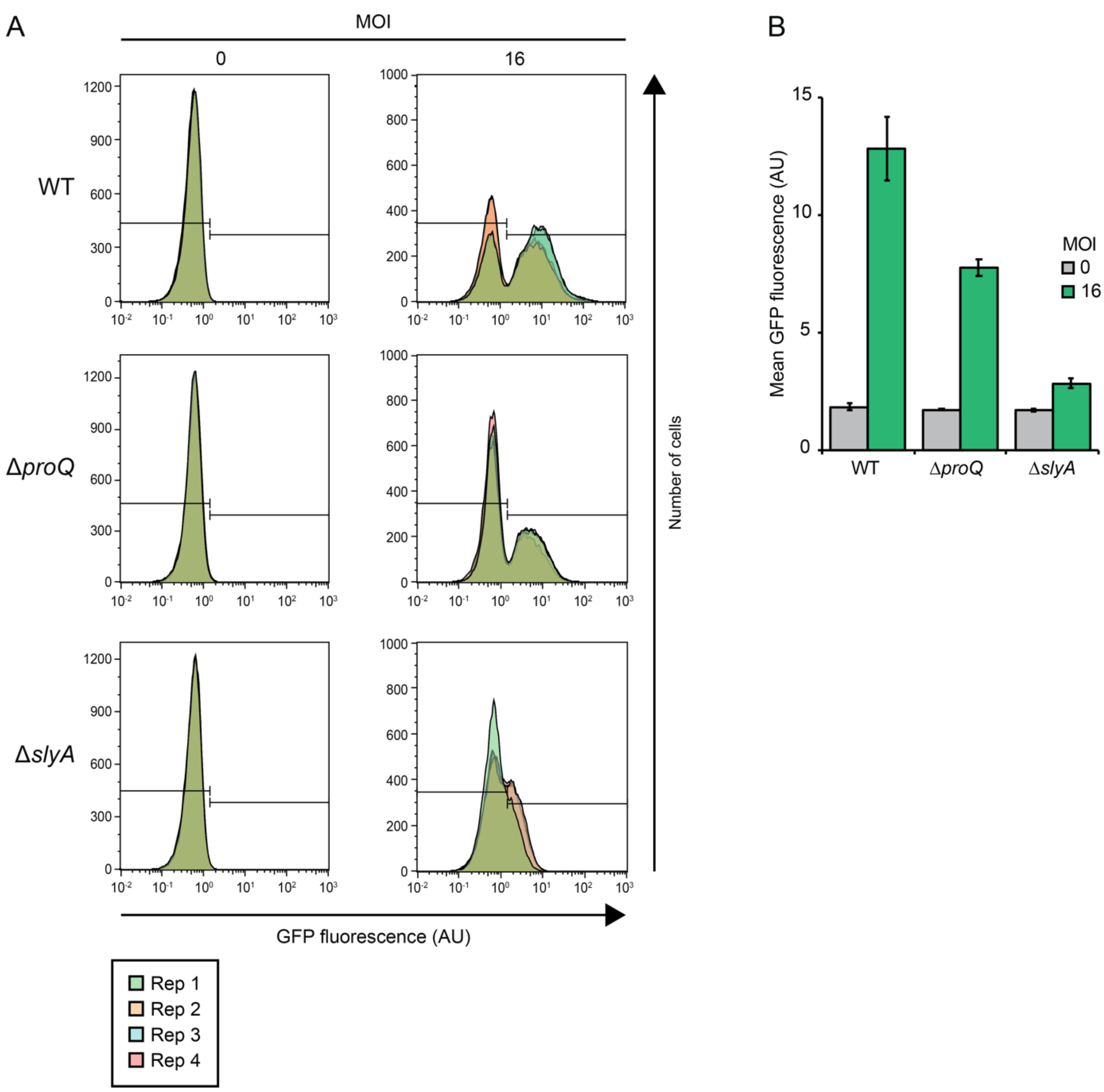

FIG S2. ProQ positively affects *ssaG* expression during infection of U937 cells. (A) Distribution of GFP fluorescence from U937 cells infected with the indicated *Salmonella* strains harboring a P_{ssaG}-GFP reporter at a multiplicity of infection (MOI) of 16. MOI 0 represents uninfected controls. Colors represent independent replicate infections. (B) Mean fluorescence of the GFP positive population indicated by gates in A. Error bars represent standard deviation of four biological replicates.

Figure S3

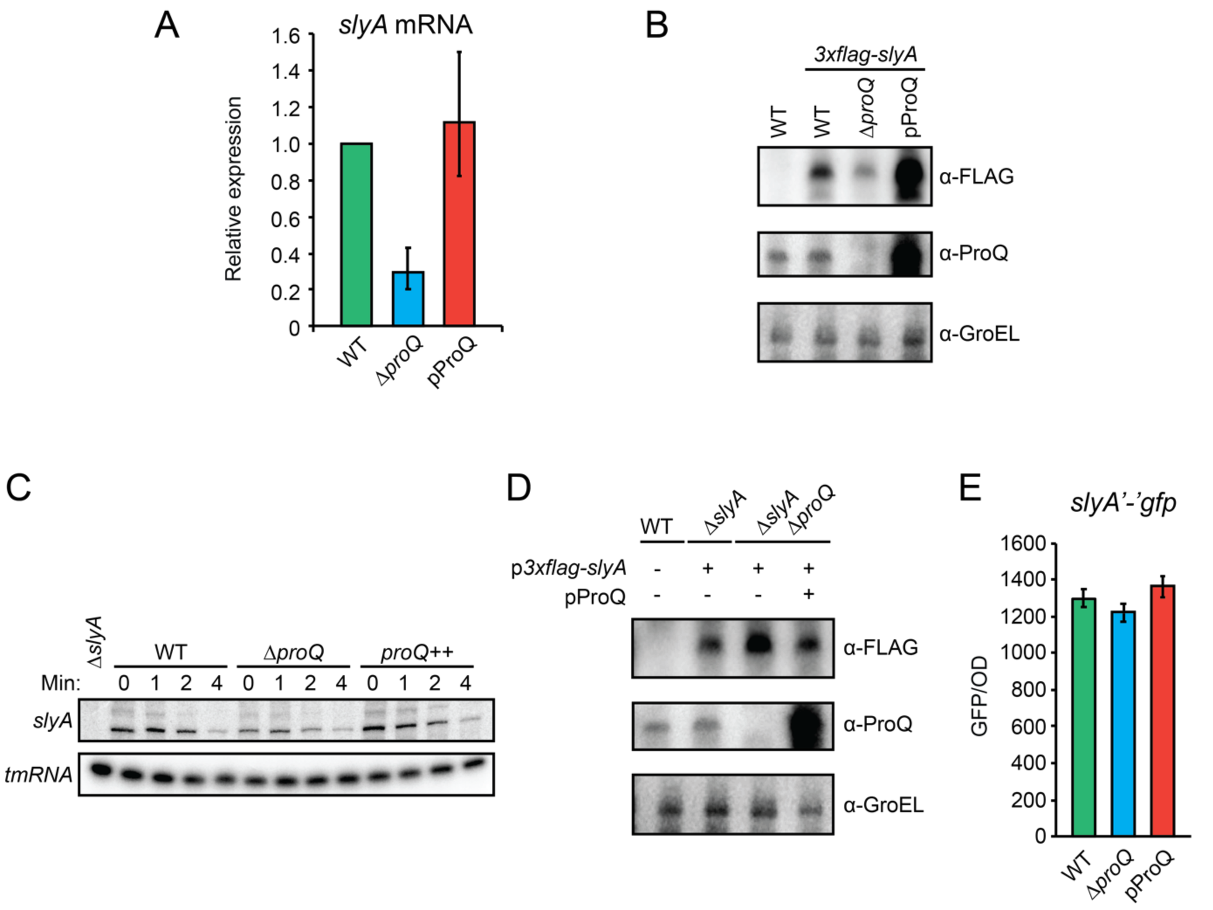

FIG S3. ProQ promotes *slyA* transcription, but not translation. (A) Steady-state levels of *slyA* mRNA as determined by qPCR for the indicated strains grown in SPI2-inducing conditions. (B) Western blot analysis of 3xFLAG-SlyA and ProQ protein levels. The 3xFLAG-SlyA protein was expressed from its native promoter on the chromosome in wild-type, Δ*proQ,* and ProQ overexpression strains. WT indicates a strain with non-tagged SlyA. GroEL served as loading control. (C) Rifampicin experiment during growth in SPI2-inducing conditions monitoring the decay of *slyA* mRNA in the indicated strains. *proQ++* indicates a Δ*proQ* strain expressing *proQ* from its native promoter on a multi-copy plasmid. A 5'-labeled oligonucleotide specific for *slyA* mRNA was used for detection. tmRNA served as loading control. (D) Western blot monitoring 3xFLAG-SlyA and ProQ protein levels. The 3xFLAG-SlyA protein was expressed from a P_{araBAD} promoter on a plasmid in Δ*slyA* or Δ*slyA*Δ*proQ* strains. The strains include either a control plasmid or a plasmid overexpressing ProQ from an IPTG-inducible promoter (pProQ). GroEL served as loading control. (E) GFP expression from a translational *slyA*-*gfp* fusion in wild-type, Δ*proQ,* and Δ*proQ* with or without ProQ overexpression from an IPTG-inducible promoter on plasmid pProQ during growth in SPI2 medium. Wild-type and Δ*proQ* strains harbor the empty vector pAR007 (pProQ backbone).

Figure S4

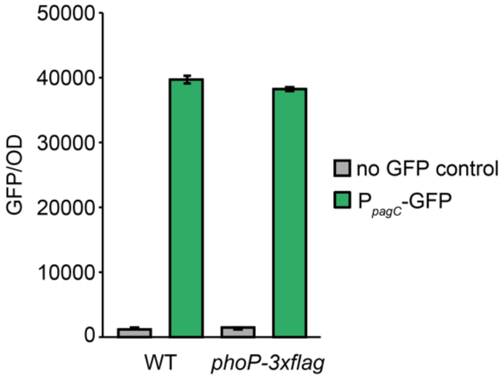

FIG S4. Addition of a C-terminal 3xFLAG tag does not affect PhoP activity. GFP expression from a P_{pagC}-*gfp* transcriptional reporter in a *Salmonella* wild-type strain and an isogenic strain harboring a chromosomal *phoP-3xflag* allele. Bacteria were grown in SPI2 medium for 18 hours. The no GFP control refers to strains harboring an empty control plasmid lacking the *gfp* gene. Error bars represent standard deviation of three biological replicates.

Table S1: CLIP-seq data, see excel file (Table S1.xlsx).

Table S2: Bacterial strains used in this study

Strain number	Strain	Specification	Selection marker	Reference
JVS-1574	SL1344			(1)
JVS-10132	SL1344	*proQ*-3x*flag*		(2)
JVS-11364	SL1344	*ΔproQ:Km*	KmR	(3)
JVS-11365	SL1344	*ΔproQ*		(3)
1867, McClelland collection	14028s	*ΔslyA:Km*	KmR	(4)
EHS-1876	SL1344	*ΔslyA:Km*	KmR	(5)
EHS-1880	SL1344	*ΔslyA*		(5)
EHS-1882	SL1344	*ΔproQΔslyA*		(5)
EHS-3257	SL1344	*PhoP 3'UTR ::KanSacB*	KmR, TetR	This study
EHS-3288	SL1344	*PhoP-3xFLAG*		This study
EHS-3316	SL1344	*PhoP-3xFLAG::ΔproQ*::Km	KmR	This study
EHS-2089	SL1344	*slyA Δ5'UTR::KanSacB*	KmR, TetR	This study
EHS-2294	SL1344	*SlyA-3xFLAG*		This study
EHS-2300	SL1344	*SlyA-3xFLAG::ΔproQ*::Km	KmR	This study

Table S3: Oligonucleotides used in this study

Oligos	Sequence (5' -> 3')	Description	Ref
EHO-1314	GTTTTTctcgagACAGCCTCATTTATTAGAGCG	Cloning pYMB005	This study
EHO-1253	GTTTTTggatccCATCGTAAGGATACTGGCAAC	Cloning pYMB005	This study
EHO-1315	GTTTTTctcgagGCGTAAAGAGGGAGAGATCC	Cloning pSB008	This study
EHO-1508	GTTTTTggatccTATAATTAGCTTGCTAAGTTATTATGC	Cloning pSB008	This study
EHO-1603	GTTTTTctcgagCTGCCTCACCCTCTTTTCTT	Cloning pSB011	This study
EHO-1604	GTTTTTggatccGTGTTAACAATAAGAACAGTCTAG	Cloning pSB011	This study
EHO-1623	GTTTTTctcgagGCGTGTTCAATATTTGCG	Cloning pSB012	This study
EHO-1624	GTTTTTggatccGTTAGTATCGGCCTGTGCA	Cloning pSB012	This study
EHO-1673	GTTTTCTCGAGCGCTAACGGATTCACCACTC	Cloning pEH809	This study
EHO-1674	GTTTTATGCATTAACTGTCAGACCAAGTTTACTC	Cloning pEH809	This study
EHO-1675	ATCGATGCATAATGTGCCTG	Cloning pEH809	This study
EHO-1676	GTTTTCTCGAGCGCCGAATAAATACCTGTGAC	Cloning pEH809	This study
EHO-1689	GTTTTGCTAGCAAAATCAGCATAATAACTTAGCAAGC	Cloning pEH811	This study
EHO-1690	GTTTTAAGCTTCAACACCATTATGGTTGTGCA	Cloning pEH811	This study
EHO-450	CGAGCATTTAAATCTAGAGGCATC	Cloning pEH791	This study
EHO-1343	GTTTTATGCATATGTGCTCAGTATCTCTATCACTG	Cloning pEH791	This study
EHO-1340	GTTTTATGCATAAAATCAGCATAATAACTTAGCAAGC	Cloning pEH791	This study
EHO-1342	GTTTTTCTAGACAACACCATTATGGTTGTGCA	Cloning pEH791	This study
EHO-1458	TTATAAAGATCATGATATCGACTACAAAGATGACGACGAT AAAGAATCGCCACTAGGTTCTGA	Cloning pSB001	This study
EHO-1459	CGATATCATGATCTTTATAATCACCGTCATGGTCTTTGTAG TCCAATTTCATCTCCTTATAATTAGCT	Cloning pSB001	This study
EHO-1463	GTTTTAAGCTTAAAATCAGCATAATAACTTAGCAAGC	Cloning pSB006	This study
EHO-1465	GTTTTCTGCAGCTCGAGGTGAAGACGAAAGG	Cloning pSB006	This study
EHO-1466	GTTTTCTGCAGCGCCGTTACCACCGCTGCGT	Cloning pSB006	This study
EHO-1467	GTTTTAAGCTTTACGACCAGTCTAAAAAGCGCCTG	Cloning pSB006	This study
EHO-1786	P- TAGACTGTTCTTATTGTTAACACAAGGGAGAAGAGATGAT GCGCGTACTGGTTGTAGAGGATAATG	Cloning pEH839	This study
EHO-1787	P- CTAGCATTATCCTCTACAACCAGTACGCGCATCATCTCTTC TCCCTTGTGTTAACAATAAGAACAGTCTATGCA	Cloning pEH839	This study
EHO-1339	GTTTTATGCATGCAATGTTCCTTTGCGTCAG	Cloning pEH843	This study
EHO-1790	GTTTTGCTAGCTGCCAGATCAGAACCTAGTG	Cloning pEH843	This study
EHO- 1363	CACCAACCGTGCCAGATCAGAACCTAGTGGCGATTCCAATT GTAGGCTGGAGCTGCTT	Cloning EHS-2294	This study
EHO-1364	GAGAATACAAATACTGCACACTATTCTAAAATCAGCATAAA CATATGAATATCCTCCTTAGTTCC	Cloning EHS-2294	This study
EHO-1869	TACCACCGTACGCGGACAAGGATATCTTTTTGAATTGCGCtg taggctggagctgcttc	Cloning EHS-3288	This study
EHO-1870	AGCGACAGCGGCAGAAAATGGCGAGCAAATTTATTCATTAa catatgaatatcctccttagttcc	Cloning EHS-3288	This study
EHO-1502	AGCAATACATTTGTTTTGAG	Cloning EHS-2294	This study
EHO-1503	GTCAATTCCAGAGGCTTG	Cloning EHS-2294	This study
EHO-1871	TACCACCGTACGCGGACAAGGATATCTTTTTGAATTGCGCG ACTACAAAGACCATGACGGTGATTATAAAGATCATGATATC G	Cloning EHS-3288	This study
EHO-1872	AGCGACAGCGGCAGAAAATGGCGAGCAAATTTATTCATTAT TTATCGTCGTCATCTTTGTAGTCGATATCATGATCTTTATA A	Cloning EHS-3288	This study

EHO-1845	TCATCGCTGAAAACAGGG	Northern blot probe for SL1344 MicA sRNA	This study
EHO-1846, JVO-4656	ACGAGCAAGCATCATATTGG	Northern blot probe for SL1344 MicL (RyeF, STnc860) sRNA	(6)
EHO-1847, JVO-1205	GTTGATGGGCTCCACAA	Northern blot probe for SL1344 RybB sRNA	(7)
EHO-1848, JVO-2408	TATGAGGAGGACAATTACCG	Northern blot probe for SL1344 PinT (STnc440) sRNA	(8)
EHO-1850, JVO-4661	CTTGATTACCACAACCACATCA	Northern blot probe for SL1344 DapZ sRNA	(6)
EHO-1851, JVO-0749	TTCGTTCCGGCTCAGGA	Northern blot probe for SL1344 GvcB sRNA (5' end)	(9)
EHO-1855	ATCCCGATGTGGTCTAGAAG	Northern blot probe for SL1344 STnc3750 sRNA	This study
EHO-1856	GTTATTCCGTGGTGACG	Northern blot probe for SL1344 STnc4210 sRNA	This study
EHO-1857	CCTCAATTCAGACCGCAAT	Northern blot probe for SL1344 STnc150 sRNA	This study
EHO-1858	CATACTCGTCCATAGTCGTG	Northern blot probe for SL1344 STnc3170 sRNA	This study
EHO-1859	AGCCAACGCAGTCAAAACGA	Northern blot probe for SL1344 STnc3110 sRNA	This study
EHO-1861	ATCTCGACGTGACAGGAA	Northern blot probe for SL1344 STnc1710 sRNA	This study
EHO-0923	GAATACTGCGCCAACACCAG	Northern blot probe for SL1344 ArcZ sRNA	This study
EHO-861	CTACGGCGTTTCACTTCTGAGTTC	Northern blot probe for SL1344 5S rRNA	This study
EHO-0913	CCGAATCCTTTGGATTCATTAAACCACTTAACGTTACCTTT	Northern blot probe for SL1344 cspE	This study
EHO-0867	TGGTGGAGCTGGCGGGAGTT	Northern blot probe for SL1344 tmRNA	This study
EHO-1125	TACCGAACATCACGCCAATC	qRT-PCR, recA	This study
EHO-1126	GTATGATGAGCCAGGCGATG	qRT-PCR, recA	This study
EHO-1082	CGTCAAGAGCGTATTTTTAGCA	qRT-PCR, pagK	This study
EHO-1083	GGCATTGGCTTAATTGTTTCTT	qRT-PCR and northern blot probe, pagK	This study
EHO-1086	ATCCGAGGGGTAAATGTGAA	qRT-PCR, pagC	This study
EHO-1087	GGAAGCCTGTCTGTCTCCAT	qRT-PCR and northern blot probe, pagC	This study
EHO-1096	CTCTTCTGCTGTTGTCGTCTG	qRT-PCR, pagM	This study
EHO-1097	CGCCAGGGAACAAACCT	qRT-PCR and northern blot probe, pagM	This study
EHO-1080	AAAGATGGTTTCGGGAGGTAA	qRT-PCR, pipB	This study
EHO-1081	CGTATTTTCGGATGCTGCT	qRT-PCR and northern blot probe, pipB	This study
EHO-1243	GCTAAAATGATGGGCGGTAA	qRT-PCR, ssrA	This study
EHO-1244	GGCGGCTGGTATTCTTGTAA	qRT-PCR, ssrA	This study
EHO-1371	GTAATGATTTGCTCAACCCAGA	qRT-PCR, ssaG	This study
EHO-1372	TCATTTTGATCAGTGAACTTTCGT	qRT-PCR, ssaG	This study
EHO-1238	GACCTCTTCCATCTCAGCGATCAGCGGCTCCGCTTTTT	Northern blot probe for SL1344 slyA	This study
EHO-1239	ATAGGCATTGAGCAGCCATC	qRT-PCR, slyA	This study
EHO-1240	CCGCTTTTTCGGTCAGTTTA	qRT-PCR, slyA	This study

Table S4: Plasmids used in this study

Plasmid	Description	Parental plasmid	Selection marker	Reference
pJV300	Empty vector		AmpR	(10)
pZE12-proQ	*proQ*	PJV300	AmpR	(3)
pCP20	Removal of antibiotic resistance cassette		CmR	(11)
pAR007	Empty vector; PLlacO-C promoter and LacI repressor		TetR	(12)
pAR009	IPTG-inducible ProQ	pAR007	TetR	(12)
pAR011	Vector with dTomato	pAR007	TetR	(12)
pAR018	IPTG-inducible dTomato-proQ fusion	pAR011	TetR	(12)
pAR032	IPTG-inducible dTomato-proQ fusion mutation L34Q	pAR018	TetR	(12)
pAR033	IPTG-inducible dTomato-proQ fusion mutation R80H	pAR018	TetR	(12)
pAR036	IPTG-inducible dTomato-proQ fusion mutation T200P	pAR018	TetR	(12)
pAR037	IPTG-inducible dTomato-proQ fusion mutation G185V	pAR018	TetR	(12)
pUA66	Empty vector, promotorless; *gfp* gene		KmR	(13)
pXG-1	Empty vector; PLtetO-1 promoter		CmR	(14)
pXG10-SF	Empty vector; PLtetO-1 promoter and *gfp*		CmR	(15)
pYMB005	Transcriptional fusion P*ssaG*-gfp	pUA66	KmR	This study
pBR322			CmR	(16)
pEH791	*slyA*	pXG-1	CmR	This study
pEH809		pBAD33	CmR	This study
pEH811	SlyA-3xFLAG with pBAD	pEH809	CmR	This study
pSB001	SlyA-3xFLAG	pEH791	CmR	This study
pSB002	SlyA-3xFLAG	pYMB018	CmR	This study
pSB006	Rha promoter SlyA-3xFLAG	pSB001	CmR	This study
pSB008	Transcriptional fusion P*slyA*-gfp	pUA66	KmR	This study
pSB011	Transcriptional fusion P*phoP*-gfp	pUA66	KmR	This study
pSB012	Transcriptional fusion P*pagC*-gfp	pUA66	KmR	This study
pEH839	Translational fusion *phoP* 5’UTR	pXG10-SF	CmR	This study
pEH843	Translational fusion *slyA* 5’UTR	pXG10-SF	CmR	This study
pSlm5-tet	Temperature-controlled λ-red system; Lambda red recombination		TetR	(17)
pSCrhaB2	Empty vector, rhamnose-inducible promoter		TpR	(18)

Supplementary references

1. Hoiseth SK, Stocker BAD. 1981. Aromatic-dependent Salmonella typhimurium are non-virulent and effective as live vaccines. Nature 291:238–239.
2. Holmqvist E, Li L, Bischler T, Barquist L, Vogel J. 2018. Global Maps of ProQ Binding In Vivo Reveal Target Recognition via RNA Structure and Stability Control at mRNA 3′ Ends. Mol Cell 70:971-982.e6.
3. Smirnov A, Förstner KU, Holmqvist E, Otto A, Günster R, Becher D, Reinhardt R, Vogel J. 2016. Grad-seq guides the discovery of ProQ as a major small RNA-binding protein. Proc Natl Acad Sci U S A 113:11591–11596.
4. Porwollik S, Santiviago CA, Cheng P, Long F, Desai P, Fredlund J, Srikumar S, Silva CA, Chu W, Chen X, Canals R, Reynolds MM, Bogomolnaya L, Shields C, Cui P, Guo J, Zheng Y, Endicott-Yazdani T, Yang HJ, Maple A, Ragoza Y, Blondel CJ, Valenzuela C, Andrews-Polymenis H, McClelland M. 2014. Defined single-gene and multi-gene deletion mutant collections in salmonella enterica sv typhimurium. PLoS One 9:e99820.
5. Rizvanovic A, Michaux C, Panza M, Iloglu Z, Helaine S, Wagner GEH, Holmqvist E. 2022. The RNA-Binding Protein ProQ Promotes Antibiotic Persistence in Salmonella. MBio 13.
6. Chao Y, Papenfort K, Reinhardt R, Sharma CM, Vogel J. 2012. An atlas of Hfq-bound transcripts reveals 3′ UTRs as a genomic reservoir of regulatory small RNAs. EMBO J 31:4005–4019.
7. Papenfort K, Pfeiffer V, Mika F, Lucchini S, Hinton JCD, Vogel J. 2006. σE-dependent small RNAs of Salmonella respond to membrane stress by accelerating global omp mRNA decay. Mol Microbiol 62:1674–1688.
8. Westermann AJ, Förstner KU, Amman F, Barquist L, Chao Y, Schulte LN, Müller L, Reinhardt R, Stadler PF, Vogel J. 2016. Dual RNA-seq unveils noncoding RNA functions in host–pathogen interactions. Nat 2016 5297587 529:496–501.
9. Sharma CM, Darfeuille F, Plantinga TH, Vogel J. 2007. A small RNA regulates multiple ABC transporter mRNAs by targeting C/A-rich elements inside and upstream of ribosome-binding sites. Genes Dev 21:2804.
10. Sittka A, Pfeiffer V, Tedin K, Vogel J. 2007. The RNA chaperone Hfq is essential for the virulence of Salmonella typhimurium. Mol Microbiol 63:193–217.
11. Cherepanov PP, Wackernagel W. 1995. Gene disruption in Escherichia coli: TcR and KmR cassettes with the option of Flp-catalyzed excision of the antibiotic-resistance determinant. Gene 158:9–14.
12. Rizvanovic A, Kjellin J, Söderbom F, Holmqvist E. 2021. Saturation mutagenesis charts the functional landscape of Salmonella ProQ and reveals a gene regulatory function of its C-terminal domain. Nucleic Acids Res 49:9992–10006.
13. Zaslaver A, Bren A, Ronen M, Itzkovitz S, Kikoin I, Shavit S, Liebermeister W, Surette MG, Alon U. 2006. A comprehensive library of fluorescent transcriptional reporters for Escherichia coli. Nat Methods 3:623–628.
14. Urban JH, Vogel J. 2007. Translational control and target recognition by Escherichia coli small RNAs in vivo. Nucleic Acids Res 35:1018–1037.
15. Corcoran CP, Podkaminski D, Papenfort K, Urban JH, Hinton JCD, Vogel J. 2012. Superfolder GFP reporters validate diverse new mRNA targets of the classic porin regulator, MicF RNA. Mol Microbiol 84:428–445.
16. Bolivar F, Rodriguez RL, Greene PJ, Betlach MC, Heyneker HL, Boyer HW, Crosa JH, Falkow S. 1977. Construction and characterization of new cloning vehicle. II. A multipurpose cloning system. Gene 2:95–113.

17. Koskiniemi S, Pränting M, Gullberg E, Näsvall J, Andersson DI. 2011. Activation of cryptic aminoglycoside resistance in Salmonella enterica. Mol Microbiol 80:1464–1478.
18. Cardona ST, Valvano MA. 2005. An expression vector containing a rhamnose-inducible promoter provides tightly regulated gene expression in Burkholderia cenocepacia. Plasmid 54:219–228.
